# Lgr5^+^ stem and progenitor cells reside at the apex of a heterogeneous embryonic hepatoblast pool

**DOI:** 10.1242/dev.174557

**Published:** 2019-06-12

**Authors:** Nicole Prior, Christopher J. Hindley, Fabian Rost, Elena Meléndez, Winnie W. Y. Lau, Berthold Göttgens, Steffen Rulands, Benjamin D. Simons, Meritxell Huch

**Affiliations:** 1 The Wellcome Trust/Cancer Research UK Gurdon Institute, University of Cambridge, Tennis Court Road, Cambridge, CB2 1QN, UK; 2 The Cavendish Laboratory, Department of Physics, University of Cambridge, JJ Thompson Avenue, Cambridge, CB3 0HE, UK; 3 Max Planck Institute for the Physics of Complex Systems, Nöthnitzer Strasse 38, 01187 Dresden, Germany; 4 Department of Haematology and Wellcome and MRC Cambridge Stem Cell Institute, University of Cambridge, Cambridge, CB2 0XY, UK; 5 Center for Systems Biology Dresden, Pfotenhauer Strasse 108, 01307 Dresden, Germany; 6 Wellcome Trust - Medical Research Council Cambridge Stem Cell Institute, University of Cambridge, Tennis Court Rd, Cambridge, CB2 1QR, UK; 7 Department of Physiology, Development and Neuroscience, University of Cambridge, Cambridge, CB2 3DY, UK

**Keywords:** Hepatoblast, Lgr5, Organoid, Bipotent, Liver stem/progenitor cells, Liver development

## Abstract

During mouse embryogenesis, progenitors within the liver known as hepatoblasts give rise to adult hepatocytes and cholangiocytes. Hepatoblasts, which are specified at E8.5-E9.0, have been regarded as a homogeneous progenitor population that initiate differentiation from E13.5. Recently, scRNA-seq analysis has identified sub-populations of transcriptionally distinct hepatoblasts at E11.5. Here, we show that hepatoblasts are not only transcriptionally but also functionally heterogeneous, and that a subpopulation of E9.5-E10.0 hepatoblasts exhibit a previously unidentified early commitment to cholangiocyte fate. Importantly, we also identify a subpopulation constituting 2% of E9.5-E10.0 hepatoblasts that express the adult stem cell marker *Lgr5*, and generate both hepatocyte and cholangiocyte progeny that persist for the lifespan of the mouse. Combining lineage tracing and scRNA-seq, we show that *Lgr5* marks E9.5-E10.0 bipotent liver progenitors residing at the apex of a hepatoblast hierarchy. Furthermore, isolated Lgr5^+^ hepatoblasts can be clonally expanded *in vitro* into embryonic liver organoids, which can commit to either hepatocyte or cholangiocyte fates. Our study demonstrates functional heterogeneity within E9.5 hepatoblasts and identifies *Lgr5* as a marker for a subpopulation of bipotent liver progenitors.

## Abstract (precis)

**Summary:** Multicolour clonal genetic lineage tracing, organoid cultures and scRNA-seq analyses demonstrate that Lgr5 marks a subpopulation of bipotent hepatoblasts that reside at the apex of the hierarchy of a heterogenous hepatoblast pool.

## INTRODUCTION

The liver is composed predominantly of hepatocytes and cholangiocytes [also known as ductal cells or biliary epithelial cells (BECs)]. These epithelial cells work in conjunction with the liver stromal, endothelial and mesenchymal cells to perform essential metabolic, exocrine and endocrine functions (Zorn, 2008). In addition, the epithelial cells have a tremendous capacity for regeneration, which is vital given the constant exposure of the liver to metabolic and toxic substances.

During mouse embryogenesis, liver specification from the ventral foregut endoderm begins at embryonic day (E)8.5, followed by the formation of the hepatic diverticulum. Circa E9.5, hepatic endoderm cells, termed hepatoblasts, proliferate, delaminate and migrate into the adjacent septum transversum mesenchyme (STM) to form the liver bud. Hepatoblasts are the embryonic progenitors for adult hepatocytes and cholangiocytes, whereas the STM contributes to the prospective hepatic mesenchyme (Medlock and Haar, 1983; Zorn, 2008). The STM and hepatic mesenchyme secrete several growth factors, including FGF, BMP, HGF and Wnt, which promote hepatoblast proliferation, migration and survival (reviewed by Zorn, 2008). Histological data at E13.5 show subsets of hepatoblasts near the portal mesenchyme upregulate biliary-specific cytokeratins, indicating that biliary differentiation is initiated by E13.5 (Germain et al., 1988; Lemaigre, 2003). By contrast, hepatoblasts that are not in contact with portal veins respond to signals from the closely associated haematopoietic cells in the liver and differentiate into hepatocytes (Zorn, 2008).

Previous studies have hinted at the bipotential nature of hepatoblasts; immunohistochemical analysis in rats showed that the expression of proteins such as γ-glutamyl transpeptidase, which is detected at low levels in almost all hepatoblasts, becomes upregulated and restricted to differentiated cholangiocytes only and not to hepatocytes (Germain et al., 1988). Similarly, hepatoblasts near the portal mesenchyme, which are destined to become cholangiocytes, transiently express *Afp* and *Alb*, two markers that later become restricted to hepatocytes (Shiojiri et al., 2001). These reports show that the hepatoblast population expresses markers of both hepatocytes and cholangiocytes, which later become lineage restricted. More recent studies have used positive selection with surface markers to isolate hepatoblasts before characterisation (as reviewed by Miyajima et al., 2014). However, the processes that regulate the cholangiocyte versus hepatocyte decision remain unclear. It is also unclear whether a single hepatoblast can give rise to both cholangiocytes and hepatocytes, i.e. whether single hepatoblasts are bipotent or whether there are subpopulations of unipotent hepatoblasts.

During endoderm patterning, Wnt signalling represses liver fate (McLin et al., 2007), but is required at E10 for liver bud formation (Micsenyi et al., 2004) and hepatic proliferation (Tan et al., 2008). The Wnt target gene *Lgr5* was originally described as an adult intestinal stem cell marker (Barker et al., 2007). *Lgr5* has since been reported to be a marker of cycling adult stem cells in many other organs, such as the stomach, mammary gland and tongue, among others (Koo and Clevers, 2014). In the homeostatic liver, *Lgr5* expression is restricted to pericentral hepatocytes (Planas-Paz et al., 2016). However, in response to damage, *Lgr5* expression becomes highly upregulated (Huch et al., 2013) and mice lacking both *Lgr5* and its homologue *Lgr4* show impaired proliferation in pericentral (Planas-Paz, 2016) and periportal hepatocytes (Karaca et al., 2014). In the embryo, *Lgr5* has been reported as a marker of bipotent progenitors in developing mammary cells (Trejo et al., 2017), kidney (Barker et al., 2012) and intestine (Kinzel et al., 2014). Bulk RNA-seq analysis of embryonic tissue has identified many components of the Wnt pathway, including *Lgr5*, to be differentially expressed in the E10.5 liver compared with the embryonic pancreas (Rodríguez-Seguel et al., 2013). Furthermore, recent single cell RNA sequencing (scRNA-seq) analysis of E11.5 livers reported that the embryonic liver harbours subpopulations of transcriptionally heterogeneous hepatoblasts, some of which express *Lgr5* (Yang et al., 2017). However, these studies did not address whether the transcriptional heterogeneity observed reflects a genuine functional heterogeneity of the hepatoblast pool, nor did they investigate the role of Lgr5^+^ cells during embryonic liver development. Here, by combining multicolour clonal genetic lineage tracing, organoid cultures and scRNA-seq analysis, we demonstrate that Lgr5 marks a subpopulation of bona fide bipotent hepatoblasts that reside at the apex of a hepatoblast hierarchy.

## RESULTS

### Lgr5 is a marker of hepatoblasts in the E9.5 liver

**Fig. 1.**
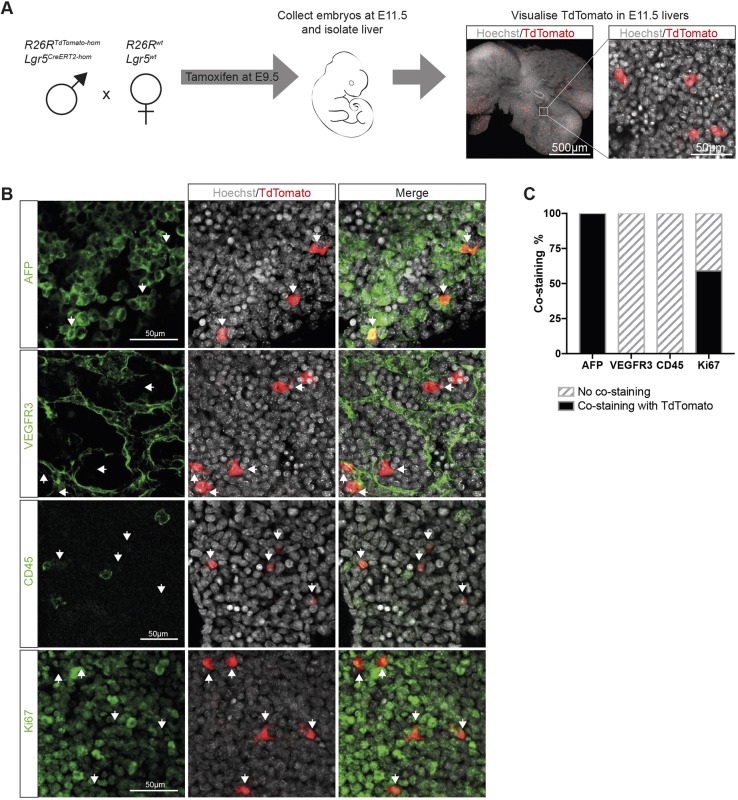
**Lgr5 expression marks cells with hepatoblast features in the developing liver.** (A-C) *Lgr5-IRES-CreERT2^hom^; R26R-TdTomato^hom^* males were mated with MF1-WT females in order to generate *Lgr5-IRES-CreERT2^het^;R26R-TdTomato^het^* embryos. Administration of tamoxifen to pregnant females at E9.5 leads to activation of Cre in Lgr5^+^ cells and recombination at the ROSA locus to induce expression of TdTomato in E9.5-E10 Lgr5^+^ cells and their progeny. (A) Schematic of experimental approach. Expression of TdTomato can be detected in E11.5 livers following induction at E9.5, indicating the presence of Lgr5^+^ cells in the developing liver at E9.5 (*n*≥3 independent experiments, *n*=2 independent litters). Representative images of TdTomato epi-fluorescence (red) are shown. Nuclei were counterstained with Hoechst (grey). (B) Representative immunofluorescent staining of TdTomato-expressing cells co-stained for the hepatoblast marker AFP (green, top panel), the endothelial marker VEGFR3 (green, middle top panel), the pan-haematopoietic marker CD45 (green, middle bottom panel) and the proliferative marker Ki67 (green, bottom panel). (C) Quantification of the immunostaining shown in B. All TdTomato^+^ cells co-express AFP and are negative for endothelial and haematopoietic fate markers (*n*>30, *n*=2 independent litters). At least half of the TdTomato^+^ cells are proliferative (Ki67^+^, *n*>50, *n*=2 independent litters).

Lgr5 expression has been reported in the developing liver as early as E10.5 (Rodríguez-Seguel et al., 2013; Yang et al., 2017). However, these studies were performed at the RNA level and there was no functional assessment of the potentiality of Lgr5-expressing cells. To investigate whether Lgr5 marks bona fide hepatoblasts, we used a lineage-tracing strategy to identify the progeny of Lgr5-expressing cells (Kretzschmar and Watt, 2012). Thus, we generated *Lgr5-IRES-CreERT2/R26R-TdTomato* embryos where, upon tamoxifen induction, *Lgr5^+^* cells and their progeny become labelled with TdTomato. As hepatoblast delamination and formation of the liver bud occurs at E9.5, we first assessed whether Lgr5 is expressed within this very early hepatoblast pool. To this end, we induced E9.5 embryos with tamoxifen and collected embryos at E11.5. We found that Lgr5 is expressed as early as E9.5-E10 (considering the time lag for tamoxifen to induce TdTomato expression) in the embryonic liver, as we detected TdTomato^+^ fluorescence in the isolated livers (Fig. 1A) and determined the labelling efficiency of Lgr5^+^ cells to be 19.6±2.2%. We next sought to address which cell type(s) express Lgr5 during liver development. We found that, at E11.5, Lgr5^+^ cells labelled at E9.5 co-expressed α fetoprotein (AFP), a well-characterised hepatoblast marker, but did not co-express markers for the endothelial (VEGFR3) or hematopoietic (CD45) lineages (Fig. 1B,C). Although labelled cells do not express endothelial markers, we found that they are located directly adjacent to the endothelial cells (Fig. 1B, Movie 1), suggesting that cell-cell interactions between the endothelium and hepatoblasts may serve to pattern the tissue. Additionally, staining with Ki67 revealed that over half of the Lgr5^+^ cells were proliferative (Fig. 1B,C). Collectively, these results reveal the existence of a population of proliferative Lgr5^+^ cells with hepatoblast features at E9.5-E10.

**Fig. 2.**
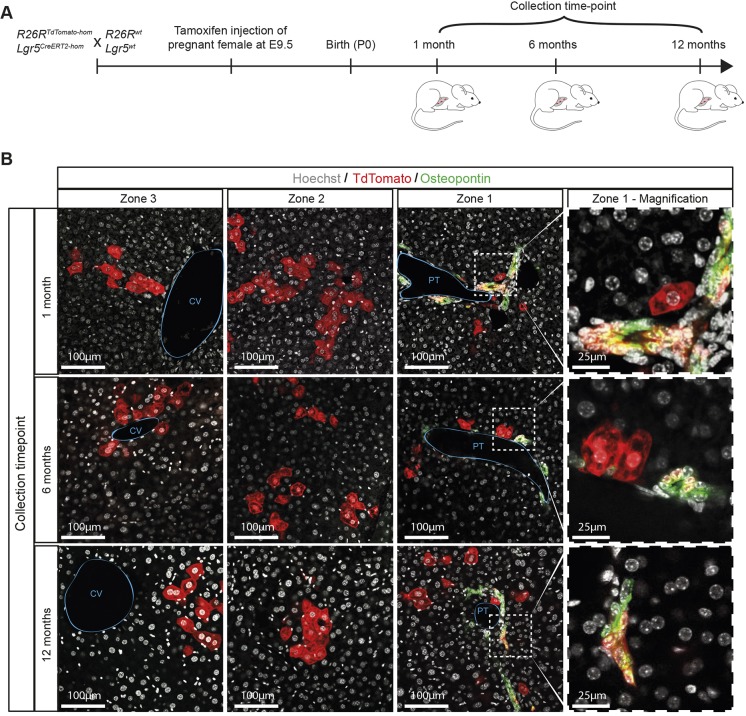
**Lgr5 is a marker of bona fide hepatoblasts *in vivo*.** (A) Schematic of experimental approach. TdTomato expression was induced at E9.5 and livers collected at the indicated postnatal time-points. (B) Lgr5^+^ progeny (TdTomato^+^ cells, red) are found distributed along all three zones of the liver lobule; the portal triad (PT, zone 1), the central vein (CV, zone 3) and the intermediate region (zone 2), at all time-points analysed, up to 12 months after birth. In zone 1, labelled cells include both hepatocytes and cholangiocytes (osteopontin, green), indicating that E9.5-E10 Lgr5^+^ cells are bona fide hepatoblasts. Right panels are enlargements of the boxed areas in zone 1.

To assess whether Lgr5^+^ cells are bona fide hepatoblasts, we analysed their contribution to the formation of both mature hepatocytes and cholangiocytes in the postnatal liver. We induced *Lgr5-IRES-CreERT2/R26R-TdTomato* embryos at E9.5 and collected postnatal livers over the course of a year (Fig. 2A). We detected TdTomato^+^ descendants of the initially labelled E9.5-E10 Lgr5^+^ cells at all time-points analysed (from 1 month up to 1 year after birth) in all three functional zones of the liver (zones 1-3; Fig. 2B). Importantly, we identified both hepatocytes and cholangiocytes as descendants of the E9.5 Lgr5^+^ hepatoblasts (Fig. 2B, Fig. S1A, Table S1, part 1). By contrast, induction at a later time-point (E13.5) resulted in only hepatocyte labelling, indicating that, by E13.5-E14, Lgr5^+^ liver progenitors are committed to hepatocyte fate (Fig. S1B, Table S1, part 2). Of note, induction at earlier time points (E7.5 and E8.5) did not result in any labelled progeny in the postnatal liver (Fig. S1C) suggesting that, at this stage of embryonic development, Lgr5 marks exclusively liver progenitors after specification and liver bud formation, but not definitive endoderm or foregut progenitors that will contribute to prospective liver tissue. No labelling was detected in non-induced mice (Fig. S1D). Altogether, our lineage tracing demonstrates that Lgr5 is a bona fide hepatoblast marker for E9.5-E10 liver bud hepatic progenitors with the capacity to give rise to adult hepatocytes and cholangiocytes.

### Lgr5 marks bona fide bipotent hepatoblasts at the earliest stage of embryonic liver development

**Fig. 3.**
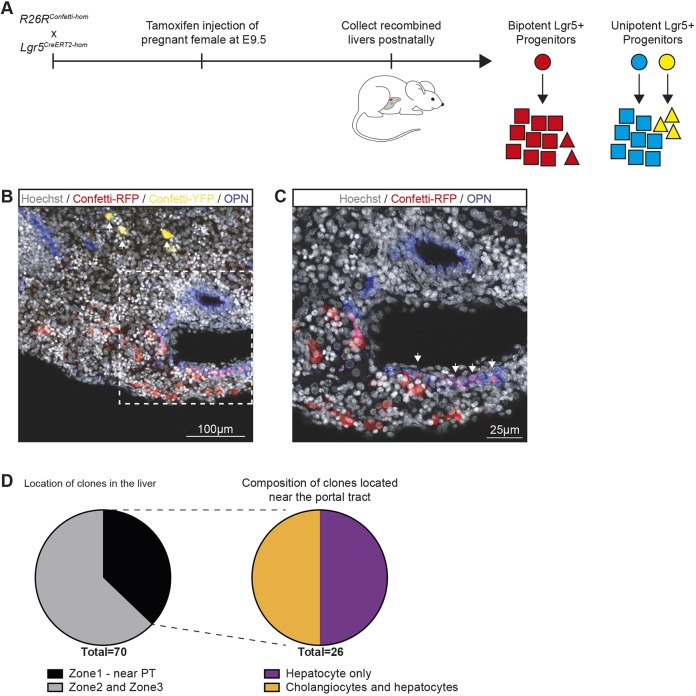
**E9.5 Lgr5^+^ hepatoblasts are bipotent.** (A-D) *Lgr5-IRES-CreERT2^hom^* mice were mated with multicolour Confetti reporter *R26R-Confetti^hom^* mice to generate *Lgr5-IRES-CreERT2^het^;R26R-Confetti^het^* embryos. Induction with tamoxifen at E9.5 results in Lgr5^+^ cells and progeny being labelled in one of four colours (RFP, YFP, mCFP or nGFP). (A) Schematic of the experimental design. Two potential outcomes are illustrated: a single Lgr5^+^ hepatoblast (red circle) is bipotent and gives rise to both hepatocytes (red squares) and cholangiocytes (red triangles); alternatively, single Lgr5^+^ hepatoblasts (blue and yellow circles) are unipotent and independently give rise to hepatocytes (blue squares) and cholangiocytes (yellow triangles). (B,C) Representative images of P0 *Lgr5-IRES-CreERT2^het^;R26R-Confetti^het^* liver following induction at E9.5. Ductal cells were co-stained for osteopontin (blue, white arrows). Nuclei were counter-stained with Hoechst. (B) Low-power magnification of a liver section showing a red and a yellow clone (white arrows). (C) Magnification showing that the red clone contains both hepatocytes and cholangiocytes (white arrows). (D) Pie charts showing the total number of clones identified (*n*=70) and the fraction of these that are located in zone 1 (*n*=26). From the total number of clones found in zone 1, half (*n*=13) contained both hepatocytes and cholangiocytes of the same colour. At the induction dose used, the frequency of mergers of clusters of the same colour is less than 3.6±1.9% for all colours (see Fig. S2B), which confirms that at least 12 of the 13 bipotent clones identified arise from a single Lgr5^+^ cell, thus demonstrating that a fraction of Lgr5^+^ cells are bipotent at E9.5. Experiments were performed in *n*=3 embryos.

To date, it has been unclear as to whether hepatoblasts are bipotent, i.e. a single hepatoblast can give rise to both cholangiocytes and hepatocytes, or are unipotent, implying the co-existence of progenitors restricted to either hepatocyte-only or cholangiocyte-only fates. To assess whether E9.5-E10 Lgr5^+^ hepatoblasts are bipotent, we turned to a clonal lineage tracing strategy to determine the contribution of each marked cell to a given fate. To mitigate the effects of cell dispersion during development, we opted to use a multicolour lineage-tracing approach, where clones derived from single cells are labelled with different colours. Lineage tracing with the R26R-Confetti reporter (Snippert et al., 2010) in combination with the *Lgr5-IRES-CreERT2* allele results in stochastic labelling of Lgr5^+^ cells with either RFP, YFP, GFP or CFP following induction with tamoxifen at E9.5 (Fig. 3A). As expected, we detected distinct clones labelled with one of the four fluorescent proteins at all time-points analysed (P0-P17) (Fig. 3B, Fig. S2A).

Recently, we showed that, owing to cell rearrangement during the expansion of developing tissues, marked cells can become dispersed and clones ‘fragmented’, with the potential to confound the interpretation of labelling data during development (Rulands et al., 2018). Therefore, to ensure that only cells within individual clones were scored, we opted to take into consideration only those clones in the portal tract labelled with a single colour where ductal cells and hepatocytes were juxtaposed. We scored 70 individual clones in this manner, 81% of which comprised hepatocytes only. No cholangiocyte-only clones were found. Crucially, from all clones identified, 37% were identified near a portal tract, and half of these (50%) contained both labelled hepatocytes and cholangiocytes of the same Confetti colour (Fig. 3C,D, Movie 2, Table S1, part 3). As, expected, we found clones in zones 2 and 3 throughout the liver formed of hepatocytes only. Clone merger, i.e. the frequency at which labelled cells with the same colour are counted as a single clone but originate from two recombination events, was estimated to be less than 3.6±1.9% for all colours (Fig. S2A,B), indicating that, from the 13 bipotent clones identified, at least 12 clones are truly bipotent. As before, no labelling was detected in non-induced mice (Fig. S2C). Therefore, clonal analysis of individual Lgr5^+^ hepatoblasts demonstrates that, at E9.5-E10, at least some of the *Lgr5*-expressing cells are bipotent.

### Lgr5^+^ embryonic liver cells grow into organoids *in vitro* and generate both ductal- and hepatocyte-fated organoids

Single Lgr5^+^ cells isolated from the livers of adult mice (Huch et al., 2013) and humans (Huch et al., 2015) can be grown clonally into cholangiocyte-like liver organoids, which retain the bipotential characteristics of adult cholangiocyte progenitors, being able to self-duplicate while maintaining the capacity to differentiate into both hepatocytes and cholangiocytes *in vitro*. We therefore sought to assess whether Lgr5^+^ embryonic liver hepatoblasts could also form self-renewing organoids while retaining their bipotential characteristics *in vitro* by isolating Lgr5^+^ hepatoblasts and placing them in culture.

Recently, further optimization of our protocols to expand human adult liver cells (Huch et al., 2015) has facilitated the expansion of human embryonic (week 11-20 human gestation) and adult mouse liver tissue as 3D organoid cultures (Hu et al., 2018; Peng et al., 2018). However, the media requirements to establish mouse embryonic liver organoids have not yet been reported. Hence, we first sought to establish culture conditions that would enable the expansion of mouse organoid cultures from the embryonic liver, opting first to use whole liver tissue isolated from E10.5-E11.5 mouse embryos without selection for specific hepatoblast cells. The use of E10.5-E11.5 rather than E9.5 embryos was for practical reasons; at E9.5 the prospective liver has not yet formed a clear organ structure and therefore it was not possible to isolate liver alone, resulting in contamination from other foregut-derived tissues, especially stomach (data not shown). To establish cholangiocyte-like organoids from the embryonic liver, we modified our previously published protocol to expand mouse adult ductal liver organoids (Broutier et al., 2016; Huch et al., 2013) by adding a TGFβ inhibitor and forskolin (Fig. S3A) to the medium. In parallel, to establish hepatocyte-like organoids, we adapted a recently published protocol for human embryonic liver (Hu et al., 2018) by removing FGF7 during passaging (Fig. S3B). Using these culture conditions, we could expand mouse embryonic liver organoids for up to five passages (3 months in culture) (Fig. S3C).

**Fig. 4.**
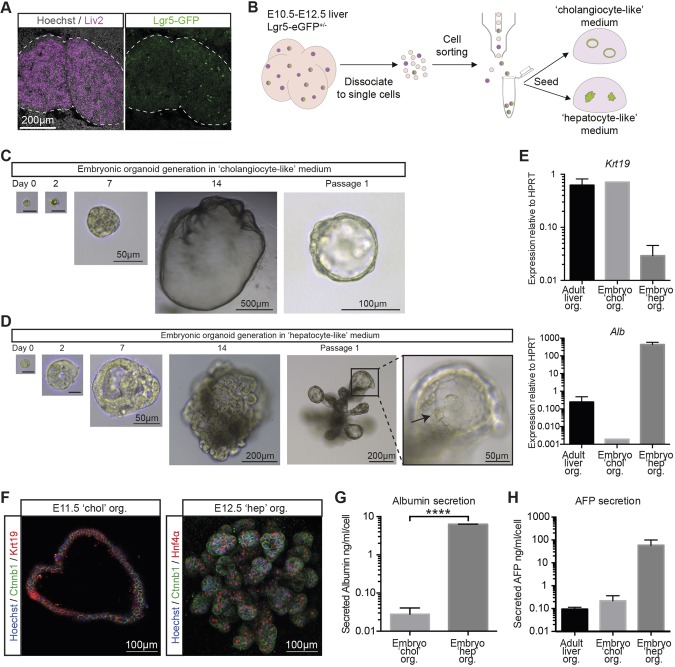
**Lgr5^+^ embryonic liver cells form both cholangiocyte-like and hepatocyte-like organoids *in vitro*.** (A) Section of an E10.5 liver showing co-labelling of Lgr5-GFP^+^ cells (green) with the liver progenitor marker Liv2 (purple). Nuclei were counterstained with Hoechst. (B-H) Embryonic liver organoids were generated from sorted Lgr5-GFP^+^ hepatoblasts isolated from E10.5-E12.5 *Lgr5-EGFP-IRES-creERT2* livers. (B) Schematic of the experimental approach. (C,D) Representative image of a mouse embryo liver organoid derived from a single Lgr5-GFP^+^ cell and cultured in (C) cholangiocyte-like medium or (D) hepatocyte-like medium. Scale bars: 10 µm in day 0 and day 2 images. The cells in the organoids grown in hepatocyte-like medium have hepatocyte morphology. (E) Expression of the ductal marker *Krt19* is predominantly detected in embryonic organoids grown in cholangiocyte-like medium and in control adult ductal liver organoids, while the hepatocyte marker albumin (*Alb*) is detected at much higher levels in embryonic cells cultured in hepatocyte-like medium. (F) Immunofluorescence staining of organoids derived from Lgr5-GFP^+^ embryonic liver cells shows clear expression of either the ductal marker Krt19 in organoids grown in cholangiocyte-like medium (left panel) or the hepatocyte marker HNF4α in organoids grown in hepatocyte-like medium (right panel) (nuclei are counterstained with Hoechst, membranes are labelled with Ctnnb1). The image of embryonic organoids grown in cholangiocyte-like medium represents a projection of 6 µm; hence, giving a multiple cell layer appearance of the single cell epithelial structure. The hepatocyte-like organoids grow as solid structures, with all cells marked by HNF4α. (G) The level of albumin secreted into the supernatant collected after 24 h is significantly higher in embryonic organoids cultured with hepatocyte-like medium compared with cholangiocyte-like medium. (H) AFP secretion into the supernatant after 24 h is increased in embryonic organoids cultured with hepatocyte-like medium when compared with cholangiocyte-like medium and with adult ductal liver organoids. Data are mean±s.e.m. of *n*≥2 experiments.

Next, we assessed whether single Lgr5^+^ cells isolated from E10.5-E12.5 livers would retain their ability to differentiate into either lineage *in vitro* when cultured in our optimized cholangiocyte-like and hepatocyte-like media conditions. To this end, we first established a sorting strategy that would enable the isolation of pure populations of Lgr5^+^ E10.5-E12.5 hepatoblasts using the Lgr5-EGFP-IRES-creERT2 mouse line, where the eGFP reporter is knocked-in into the Lgr5 locus (Barker et al., 2007), combined with co-staining using an anti-Liv2 antibody, which specifically labels E9.5-E13.5 liver progenitors (Nierhoff et al., 2005; Watanabe et al., 2002). We confirmed the hepatoblast characteristics of E10.5 Lgr5^+^ liver progenitors by co-staining using anti-Liv2 antibodies (Fig. 4A). Given that the developing liver serves as the site of haematopoiesis from E10.5 until the perinatal stage (Sasaki and Sonoda, 2000), we used negative selection of the hematopoietic marker CD45 and endothelial marker CD31 to limit contamination by non-liver progenitor cells. Embryonic livers were collected at E10.5-E12.5, enzymatically digested and then fluorescence activated cell sorting (FACS) was used to isolate (Liv2^+^/CD31^−^/CD45^−^/Lgr5-GFP^+^) Lgr5^+^ hepatoblasts (Fig. 4B, Fig. S4). Sorted Lgr5^+^ cells were embedded in Matrigel (as extracellular matrix) and cultured under our two optimized media conditions. We observed that clonally derived embryonic organoids from isolated Lgr5^+^ hepatoblasts cultured with cholangiocyte-like medium (Fig. 4C) displayed a similar expansion potential to the organoids derived from whole embryonic livers (Fig. S3C). We found that clonally derived Lgr5^+^ hepatoblasts cultured with hepatocyte-like medium readily form organoid structures (Fig. 4D), albeit with a lower expansion potential than those derived from whole embryonic livers (Fig. S3C).

The morphology of structures generated was dependent on the culture medium used. Addition of cholangiocyte-like medium resulted in the generation of single-layered epithelial spheres (Fig. 4C). The duct-like morphology of embryonic organoids cultured with cholangiocyte-like medium and expression of the classic cholangiocyte marker *Krt19* (Fig. 4E,F) is reminiscent of mouse adult ductal liver organoids (Huch et al., 2013) (Fig. S3D). Conversely, Lgr5^+^ cells cultured with hepatocyte-like medium developed more compact, densely budding structures (Fig. 4D), which resemble the recently published human embryonic hepatocyte organoids (Hu et al., 2018). The hepatocyte-like nature of these cultures was confirmed by high levels of *Alb* expression (Fig. 4E) and clear detection of Hnf4a (Fig. 4F), and functionally by an increased secretion of albumin (Fig. 4G). Embryonic organoids cultured with hepatocyte-like medium secrete more AFP than both embryonic organoids cultured with cholangiocyte-like medium and adult ductal liver organoids (Fig. 4H). This suggests that embryonic hepatocyte-like organoids retain their embryonic status *in vitro*. These results confirm that Lgr5^+^ hepatoblasts retain self-renewal and differentiation capacity *in vitro*, being capable of differentiating towards both cholangiocyte and hepatocyte fates.

### scRNA-seq identifies heterogeneity within the hepatoblast population

**Fig. 5.**
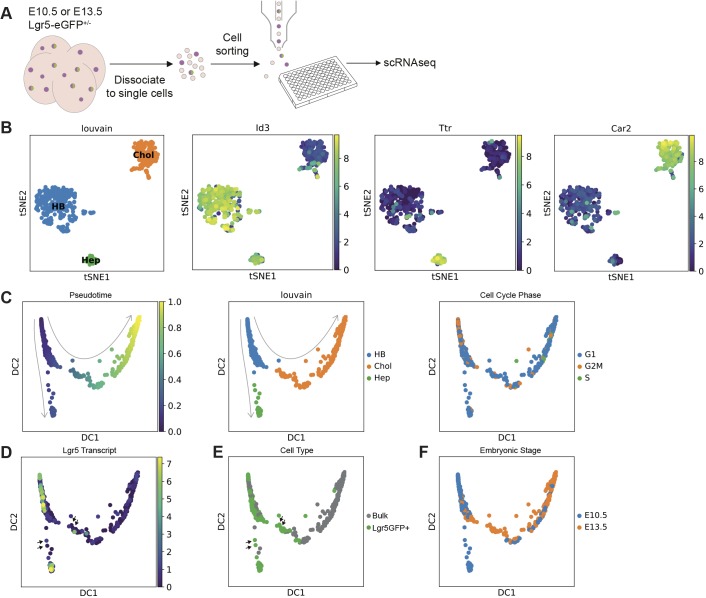
**scRNA-seq of hepatoblasts reveals heterogeneity in the hepatoblast population.** (A) Schematic of the experimental approach. Briefly, bulk (Liv2^+^) hepatoblasts and Lgr5-GFP-positive (Liv2^+^ Lgr5-GFP^+^) hepatoblasts from E10.5 or E13.5 *Lgr5-EGFP-IRES-creERT2* embryos were obtained by FACS and were processed for scRNA-seq analysis using the Smartseq2 protocol. (B) Clustering analysis (Louvain clustering) of all cells analysed (653 sorted cells from E10.5 and E13.5 embryos) classified cells into three different clusters: a cluster that exhibits features of hepatoblasts only (HB, blue), a hepatoblast cluster with hepatocyte-like features (Hep, green) and a hepatoblast cluster with cholangiocyte-like features (Chol, orange). Representative marker genes of each of these three clusters are shown: *Id3* (HB), *Ttr* (Hep) and *Car2* (Chol). Clusters are represented using tSNE plots. (C) Diffusion pseudotime analysis of E10.5 and E13.5 cells shows the HB cluster precedes the divergence of the Hep cluster or Chol cluster, and has a higher proportion of cells in G2M phase. Left panel, diffusion map showing DC1 and DC2 components; middle panel, diffusion map where the three clusters identified by Louvain clustering are shown; right panel, diffusion map showing the cell cycle phase. Arrows represent the developmental trajectory originating from the HB cluster. (D) *Lgr5* transcript levels as determined using single cell sequencing superimposed on the pseudotime analysis of all cells. (E) Lgr5-GFP^+^ cells as identified by FACS data superimposed on the pseudotime analysis of all cells. There are some Lgr5-GFP^+^ cells that were sorted as GFP^+^ but that have downregulated the *Lgr5* transcript (black arrows), indicating that these are immediate descendants of Lgr5^+^ cells. (F) Diffusion map showing the cells segregated by time-point (blue, E10.5; orange, E13.5). Cells sorted at E10.5 map to the HB cluster, to cells moving towards and located in the Hep cluster, and to cells located in the Chol cluster. At E13.5, the sorted cells map to the HB cluster, to the cells moving towards the Chol cluster and to the Chol cluster. Sorted cells no longer map to the Hep cluster, which may indicate that hepatocyte-committed hepatoblasts do not express the Liv2 epitope at E13.5.

To address whether all hepatoblasts express Lgr5 or whether Lgr5 is instead a marker of a specific subpopulation of bona fide bipotent hepatoblasts, we performed single cell RNA sequencing (scRNA-seq) analysis on both Lgr5^+^ hepatoblasts and bulk embryonic liver progenitors derived from either E10.5 or E13.5 livers. To isolate liver progenitors (Liv2^+^) and Lgr5^+^ hepatoblasts (Liv2^+^Lgr5^+^), we applied our established sorting strategy to E10.5 and E13.5 embryonic livers derived from Lgr5-EGFP-IRES-creERT2 mice (Fig. 5A, Fig. S4). Sorted Liv2^+^/CD31^−^/CD45^−^ cells (Liv2^+^ bulk hepatoblast population) and Liv2^+^/CD31^−^/CD45^−^/Lgr5-GFP^+^ cells (Lgr5^+^ hepatoblasts) were subjected to scRNA-seq analysis based on the Smart-seq2 protocol (Picelli et al., 2014). scRNA-seq analysis was conducted on 943 sorted cells. Following quality control, 653 cells were processed for further analysis. To reduce technical variability between biological replicates, we applied batch effect correction by matching mutual nearest neighbours.

To define embryonic liver progenitor populations, we performed dimensionality reduction using t-distributed stochastic neighbour embedding (tSNE) analysis on all 653 cells (Fig. 5B). This identified three distinct progenitor populations, which were confirmed by Louvain clustering. These three clusters signified biological differences, as each cluster contained cells from each biological replicate. The biological differences were confirmed by the expression of distinct marker genes (Fig. 5B, Fig. S5A, Table S2). The cell type identity of each cluster was assigned based on examining marker genes and comparing them with publicly available gene expression patterns in human or mouse liver (Broutier et al., 2017; Camp et al., 2017; Yang et al., 2017). We found that the three clusters corresponded to proliferating hepatoblasts (HBs), hepatocyte-like progenitors (Heps) and cholangiocyte-like progenitors (Chols), which express higher levels of representative markers. The HB cluster contained *Id3*, *Mdk* and *Gpc3*, all described as hepatoblast markers (Su et al., 2017; Yang et al., 2017), while the Hep cluster contained *Ttr*, *Alb*, *Apoa1*, *Apoa2* and *C3*, all known hepatocyte markers, and the Chol cluster expressed the ductal cell genes *Car2*, *Cd44* and *Bcl11a* (Yang et al., 2017) (Fig. 5B, Table S1). Of note, within the E10.5 Chol cluster we found two sub-clusters (Table S2, part 7). As well as identifying known cholangiocyte and hepatocyte markers, new markers for these clusters were also revealed by our analysis (Fig. S5A, Table S2).

To establish developmental trajectories between the different cells of the three clusters, we calculated diffusion maps and diffusion pseudotime. This analysis revealed a developmental trajectory originating from the HB cluster, which bifurcated towards either the Hep cluster or Chol cluster (Fig. 5C). We found the HB cluster contained a higher proportion of cells in G2M phase, indicating an increased number of proliferative cells (Fig. 5C, Fig. S5B). When analysing the lineage trajectories, we took advantage of the Lgr5-EGFP-IRES-creERT2 mouse line (Barker et al., 2007), which enabled us to identify cells that expressed *Lgr5* RNA via sequencing and determine whether they were GFP^+^ during FACS (Fig. 5D,E). As the GFP protein is more stable than the transcript, we used the comparison between the Lgr5-GFP^+^ sorted cells and the cells expressing *Lgr5* transcript as a proxy to identify the immediate descendants of Lgr5^+^ cells in the scRNA-seq population. Notably, most of the Lgr5^+^ cells mapped to the HB cluster, representing 2% of the total number of Liv2^+^ hepatoblasts at E10.5 (Fig. S5C). Interestingly, we observed that, as cells exit the HB cluster and become committed to either of the two epithelial lineages, *Lgr5* transcript levels decrease (Fig. 5D, black arrows). Many of these transitioning cells (Fig. 5E, black arrows) were negative for *Lgr5* transcript but positive for GFP, indicating that these cells have only recently reduced *Lgr5* levels as the GFP protein has not yet degraded and so can be considered immediate descendants of the Lgr5^+^ pool. Finally, once cells have transitioned to the Hep cluster, *Lgr5* is upregulated, while *Lgr5* expression is not reinitiated in the Chol cluster.

Segregation of the data by embryonic stage shows that E10.5 cells contribute to the HB cluster, the intermediate cells that are moving from the HB cluster towards the Hep cluster, the Hep cluster and cells located at the far end of the Chol cluster (Fig. 5F). At E10.5, though, we find very few cells in the transition between the HB and Chol clusters. However, some of them were Lgr5GFP^+^ that had downregulated *Lgr5* transcript, suggesting that they were immediate descendants of the E10.5 Lgr5^+^ HB cluster cells. Cells occupying this intermediate space were readily identified at E13.5, the majority of which also appear to have recently downregulated *Lgr5*, again indicating that they were immediate descendants of the Lgr5^+^ cells of the HB cluster. This implies that the proliferating Lgr5^+^ hepatoblasts do indeed give rise to cholangiocytes at E10.5, but with a higher proportion at E13.5. Intriguingly, E13.5 cells (both Lgr5GFP^+^ and bulk) contributed significantly to the Chol cluster, but we did not find E13.5 cells that mapped to the Hep cluster (Fig. 5F, Fig. S5D). This result was in striking disagreement with our knowledge of liver development and our E13.5 lineage-tracing results from the *Lgr5-IRES-CreERT2* allele, which provided evidence that E13.5 Lgr5^+^ tracing results in labelling of only hepatocytes (Fig. S1B). This indicates that, at E13.5, cells committed to a hepatocyte fate are indeed present and express *Lgr5*. Our interpretation of this discrepancy between the lineage-tracing and the scRNA-seq data is that the cells along the hepatocyte trajectory from E13.5 no longer express the epitope for the anti-Liv2 antibody used during FACS, and thus were not subject to sequencing.

Together, our scRNA-seq analysis suggested that the E10.5 embryonic liver harbours distinct subpopulations of liver progenitors that co-exist within the hepatoblast pool; an Lgr5^+^ subpopulation that contributes to both hepatocytes and cholangiocytes, and a previously unrecognized subpopulation of cells that has already downregulated *Lgr5* and started its specification to cholangiocyte fate.

### Lgr5 marks the apex cells within an E9.5 heterogeneous hepatoblast pool

**Fig. 6.**
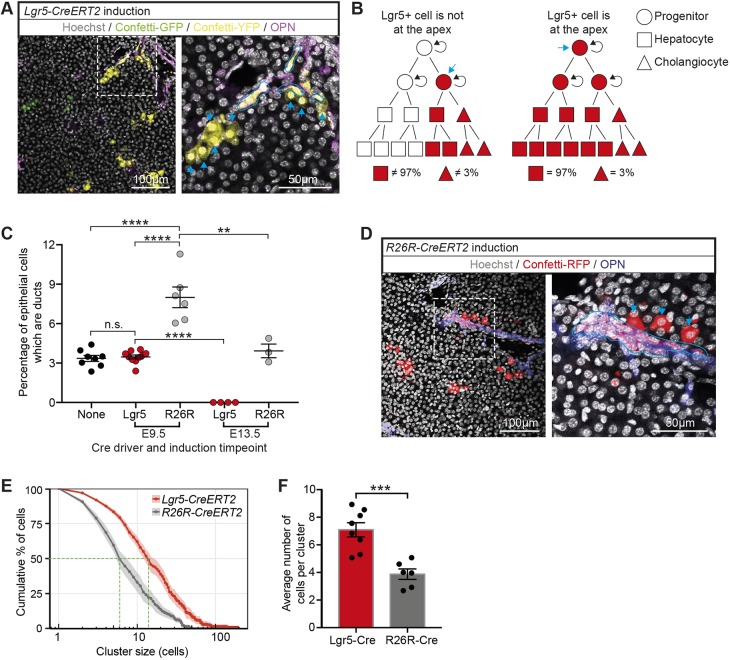
**Lgr5^+^ hepatoblasts are at the apex of the hepatoblast hierarchy.** (A-F) Embryos expressing either the Lgr5-Cre (*Lgr5-IRES-CreERT2*) or the ubiquitous Cre (*R26R-CreERT2*) were induced at E9.5 or E13.5 in order to lineage trace the early hepatoblast pool. (A) Representative images of Confetti-labelled descendants following tamoxifen induction in *Lgr5-IRES-CreERT2;R26R-Confetti* embryos at E9.5 and liver collection at P17. The magnified area highlights cholangiocytes (osteopontin, purple) outlined in blue. Arrows indicate hepatocyte clones of the same colour (yellow) as the adjacent ductal clone marked by OPN, thus indicating that they share a clonal origin. (B) Schematic displaying the possible outcomes of labelled proportions following lineage tracing of Lgr5^+^ cells at E9.5 depending on where Lgr5^+^ cells are in the hepatoblast hierarchy (indicated with a blue arrow). If E9.5 Lgr5^+^ hepatoblasts are at the apex of the hepatoblast hierarchy, it is expected that their contribution to the postnatal liver will recapitulate the homeostatic proportions of hepatocytes and ductal cells (97% versus 3%) as detailed in Fig. S6D. In the left panel, Lgr5^+^ cells (blue arrow) are not at the apex; hence, the homeostatic proportions are not achieved. By contrast, in the right panel, Lgr5^+^ cells are at the apex and therefore generate the homeostatic proportions. (C) Quantification of labelled epithelial cells following induction at E9.5 from *Lgr5-IRES-CreERT2* shows that 3.5%±0.5% were ductal, which is equivalent to the homeostatic proportion of ductal cells in the postnatal liver (no Cre driver). In contrast, the proportion of labelled ductal cells using *R26R-CreERT2* at E9.5 to drive labelling was significantly higher. At E13.5, lineage tracing from the *Lgr5-IRES-CreERT2* allele resulted in no labelled ductal cells, whereas induction from the *R26R-CreERT2* allele at E13.5 resulted in the homeostatic proportion (data are mean±s.e.m., each data point represents an individual liver). Analysis of postnatal livers was conducted at time-points P0-P30; later time-points were not considered to prevent homeostatic cellular turnover confounding the data. **P<0.01; ****P<0.0001. (D) Representative images of Confetti-labelled descendants following tamoxifen induction of *R26R-CreERT2;R26R-Confetti* embryos at E9.5 with liver collection at P14. (E) Cumulative distribution of cluster size frequency at P14-P30, comparing labelled clusters derived from Lgr5^+^ cells (*Lgr5-IRES-CreERT2*) and the bulk population (*R26R-CreERT2*) induced at E9.5 (mean±s.e.m., *n*≥6). Tracing from Lgr5^+^ cells results in larger clusters than tracing from the bulk population (F), suggesting that Lgr5^+^ cells have greater proliferative potential than the bulk population at E9.5 (data are mean±s.e.m.; ***P<0.001).

Our lineage-tracing and single cell RNA-sequencing data showed that Lgr5 labels bipotent hepatoblasts that differentiate towards hepatocyte or cholangiocyte fates. This is indicative of a hepatoblast hierarchy, and suggested Lgr5 as a potential marker of cells at its apex. Quantifying the number of tracing events as well as their contribution to the postnatal tissue provides information on the potency and commitment of a given population in the developing tissue. To determine whether Lgr5^+^ cells reside at the apex of a developmental hierarchy, we reasoned that the cell composition of their clonal progeny must reflect quantitatively the corresponding proportions in tissue. Therefore, we quantified the proportion of labelled hepatocytes and cholangiocytes following lineage tracing from *Lgr5-IRES-CreERT2* at E9.5 (Fig. 6A) and compared the proportions with the representative homeostatic distributions (Fig. 6B). We found that the homeostatic proportion of hepatocytes and cholangiocytes in the mouse postnatal liver is 96.6±0.6% and 3.4±0.6%, respectively (Fig. S6A-C, Table S1, part 4), consistent with previous reports in rats (Blouin et al., 1977). Remarkably, we found that lineage tracing with *Lgr5-IRES-CreERT2* at E9.5 resulted in labelled cells in which 96.7±0.5% were hepatocytes and 3.3±0.5% were cholangiocytes: the same proportions as the homeostatic liver (Fig. 6C). Therefore, although we cannot altogether rule out the potential parallel contribution of an Lgr5^−^ cell lineage that produces differentiated progeny in proportions representative of tissue, these results strongly suggest that Lgr5 expression marks hepatoblasts that constitute the apex of the differentiation hierarchy.

When analysing our scRNA-seq data, we found that, at E10.5, there were cholangiocyte-like cells that did not express Lgr5, suggesting that there were cholangiocyte-committed hepatoblasts even at this very early time-point. To formally investigate whether this was a genuine functional heterogeneity or was only reflecting transcriptional heterogeneity at this time-point, we turned to a second lineage tracing strategy using a ubiquitous and unbiased driver: the *R26R-CreERT2* allele. Lineage tracing from *R26R-CreERT2* will label all cell types in the developing liver, including Lgr5^+^ and Lgr5^−^ hepatoblasts. No labelling was detected in non-induced mice (Fig. S2D); therefore, labelled hepatocytes and cholangiocytes in postnatal livers will represent descendants of any hepatoblasts labelled at E9.5 (Fig. 6D). Strikingly, when labelled with the unbiased *R26R-CreERT2* allele at E9.5, we found a significantly higher proportion of labelled cholangiocytes compared with the homeostatic proportion (*R26R-CreERT*, 7.7±1.9% cholangiocytes versus 3.3±0.5% with *Lgr5-IRES-CreERT2*) (Fig. 6C). These results were confirmed using two independent multicolour R26R-reporter alleles (R26R-Confetti and R26R-Rainbow) (Table S1, part 1). These findings are in agreement with our scRNA-seq data, in which we had observed that E10.5 hepatoblasts were already committed to a cholangiocyte fate.

In contrast to induction at E9.5, Cre induction from the *R26R-CreERT2* allele at E13.5 gave rise to labelled hepatocytes and cholangiocytes in homeostatic proportions (Fig. 6C, Table S1, part 2), whereas lineage tracing from the *Lgr5-IRES-CreERT2* allele at E13.5 gave rise solely to labelled hepatocytes (Fig. 6C), suggesting that Lgr5^+^ cells lose their bipotency and position in the hierarchy during developmental progression. These results indicate that hepatoblasts are not only heterogeneous in progenitor potential but their competence to generate hepatocytes and cholangiocytes changes between E9.5 and E13.5.

In addition to the identity of labelled cells, the size of labelled clusters generated from the *Lgr5-IRES-CreERT2* and *R26R-CreERT2* alleles was quantified as a proxy for the proliferative potential of the initially labelled hepatoblast. We found that tracing with the *Lgr5-IRES-CreERT2* allele at E9.5 gave rise to larger clusters of labelled cells than tracing with the *R26R-CreERT2* allele (Fig. 6E,F). The larger cluster sizes from Lgr5^+^ hepatoblasts indicate that these cells have a greater proliferative potential than, and a developmental advantage over, the bulk hepatoblast population, again suggestive of their position at the apex of the hepatoblast hierarchy.

Our combined findings lead us to conclude that the E9.5 hepatoblast population is indeed functionally heterogeneous, with Lgr5^+^ hepatoblasts residing at the apex of the E9.5 hierarchy and a population of non-Lgr5^+^ hepatoblasts exhibiting a previously unidentified early commitment to the cholangiocyte fate.

## DISCUSSION

The Wnt target gene *Lgr5* (leucine-rich-repeat-containing G-protein-coupled receptor 5) has been described as a marker of stem cells in non-damaged, self-renewing tissues, such as the intestine, stomach and hair follicles, as reviewed by Barker et al. (2010). In the adult liver, Lgr5 is expressed at low levels during homeostasis. However, upon damage, Lgr5 becomes highly upregulated in a subset of cells, which contributes to the regeneration of the tissue. Similarly, Lgr5 is also upregulated in homeostatic liver ductal cells when cultured as self-renewing bipotential liver organoids (Huch et al., 2013). Here, we have found that Lgr5 marks a previously unknown, bipotent, Lgr5^+^ population that resides at the apex of an E9.5 heterogeneous hepatoblast pool.

To date, bipotentiality of hepatoblasts has only been shown at the population level (Yanagida et al., 2016). However, at least *in vivo*, there has been no experimental proof regarding bipotentiality of individual hepatoblast cells. A recent report showed that a labelled Foxa2^+^ definitive endoderm cell induced at E7.75 gives rise to cells moving towards hepatocyte and cholangiocyte fates at E16.5, suggesting that, at least before hepatic specification at E7.75, the definitive endoderm progenitors are multipotent (El Sebae et al., 2018). Similarly, *in vitro*, Dlk^+^ embryonic liver cells at E14.5 were found to express markers of both hepatocyte and cholangiocyte lineages (Tanimizu et al., 2003), again suggestive of the bipotent nature of hepatoblast cells. However, formal proof of bipotential hepatoblasts *in vivo* has not yet been provided. Here, using lineage tracing with a multicolour reporter, we unequivocally demonstrate that E9.5 Lgr5^+^ hepatoblasts are indeed bipotent *in vivo*, as single clones consisting of cholangiocytes and hepatocytes are present at the portal triad (Fig. 3B-D, Fig. 6A). Whether Lgr5^+^ E9.5 hepatoblasts directly give rise to hepatocytes and cholangiocytes or whether a fraction passes through a ductal-like precursor state, as suggested in a recent study (Carpentier et al., 2011), remains an interesting and unresolved issue.

Alongside bipotent clones, we also found clones formed of hepatocytes without cholangiocytes throughout the liver, including in the portal region (zone 1). This can have two possible explanations: either a subset of Lgr5^+^ cells is unipotent for hepatocyte fate and others are bipotent or, alternatively, all the Lgr5^+^ cells harbour, but may not exploit, multilineage potential. The first option (only a subset is bipotent) implies that there is engrained heterogeneity within the Lgr5^+^ population regarding their potentiality. In that regard, our single cell RNA-seq data, where we find Lgr5^+^ cells in both the hepatoblast and the hepatocyte clusters, suggest that this could indeed be a plausible scenario. Alternatively, one could hypothesise that all the Lgr5^+^ cells are bipotent but depending on external signals received according to the specific position of the original Lgr5^+^ progenitor cell, they may differentiate into one or two cell types. This implies that developmental stage and local environment could be crucial in defining the final fate of a given Lgr5^+^ hepatoblast. In this regard, the fact that embryonic Lgr5^+^ cells isolated by FACS and cultured *in vitro* were sensitive to the growth factors present in the culture medium, and committed either to the cholangiocyte or hepatocyte lineage according to media composition, would support this latter argument (Fig. 4). It is tempting to speculate that, by retaining Lgr5^+^ cells at defined positions during liver growth by maintenance of a specific local environment, differentiation into cholangiocytes would occur as daughter cells exited such a niche. This is consistent with current evidence of the discontinuous growth of the liver ductal network, as reviewed by Ober and Lemaigre (2018), although there is at present no direct evidence for a role of Lgr5^+^ cells in directing liver morphogenesis.

Interestingly, lineage tracing at E13.5 from the *Lgr5-IRES-CreERT2* allele resulted in labelling of only hepatocytes (Fig. 6C, Fig. S1B), whereas E13.5 lineage tracing from the *R26R-CreERT2* allele resulted in labelling of both hepatocytes and ductal cells in homeostatic proportions (96.4% and 3.6%, respectively). These results underline the continual shift in cell potency and cell surface marker expression throughout development of the liver and are consistent with other reports in which a single cell surface marker is not adequate to define a particular cell type (hepatoblast, hepatocyte or cholangiocyte) throughout the entirety of liver development (Tanaka et al., 2009). Instead, a set of two or more cell surface markers will have to be used to define each cell type at specific stages of development. In this regard we found that, although Liv2 is indeed a good marker of the hepatoblast pool at E10.5, it is not appropriate to identify unbiased hepatoblasts at E13.5, as it seems to mark hepatoblasts already biased towards ductal fate.

In contrast to the widely accepted view that differentiation of hepatoblasts into cholangiocytes occurs from E13.5 onwards (Gordillo et al., 2015), our results provide the functional demonstration that heterogeneity already exists at E9.5. Our scRNA-seq data show that, even as early as E10.5, there is heterogeneity within the hepatoblast population, with some cells already moving towards cholangiocyte or hepatocyte fates. We identify subpopulations of hepatoblasts that express Lgr5 while other subpopulations do not. Furthermore, some of these Lgr5^−^ cells already express markers of cholangiocyte fate. Consistent with the scRNA-seq data, our functional studies that fate map E9.5 liver progenitors using lineage tracing from either *Lgr5-IRES-CreERT2* or *R26R-CreERT2* demonstrate the existence of both Lgr5+ and Lgr5- hepatoblasts already at E9.5 (Fig. 6C). Importantly, induction of lineage tracing at E9.5 using *Lgr5-IRES-CreERT2*, but not *R26R-CreERT2*, resulted in labelled postnatal hepatocytes and cholangiocytes in homeostatic proportions (97% hepatocytes versus 3% cholangiocytes), implying that Lgr5^+^ cells behave functionally as a genuine bipotent hepatoblast and are indeed at the apex of the hepatoblast hierarchy. On the contrary, unbiased labelling using the *R26R-CreERT2* model gave rise to a higher proportion of cholangiocytes at E9.5, compared with the homeostatic or Lgr5^+^ descendants, arguing in favour of an already cholangiocyte-committed hepatoblast subpopulation, which is negative for Lgr5, in the E9.5 developing liver. This result suggests that E9.5 Lgr5^+^ cells are at the apex of their hierarchy, i.e. are bipotent and equipotent, and are able to give rise to lineage-restricted ductal progenitors that downregulate Lgr5 and expand in order to contribute to the postnatal ductal pool. Although our results demonstrate that Lgr5 expression overlaps with the apex of a hepatoblast pool, they do not show functionally that Lgr5 expression defines the apex of the hepatoblast pool. Lgr5 expression could be subject to local environmental factors and just mark a subpopulation of cells that receives high Wnt signalling. Then, one could speculate that Lgr5-negative hepatoblasts with the very same potency as the Lgr5^+^ hepatoblasts also reside at the apex of their own hierarchies. This implies that populations of equally potent hepatoblasts, some of which express Lgr5 and are bipotent, co-exist at this time-point in development. If these additional hepatoblasts populations indeed exist, the nature of their identity and bipotentiality are still issues that remain to be resolved.

In summary, using a combination of lineage-tracing, organoid cultures and scRNA-seq analysis, we show that the E9.5 hepatoblast pool is heterogeneous, not only at the RNA but also at the functional level. Within the different E9.5 hepatoblast subpopulations, we find that Lgr5 marks a previously unknown, bipotent, Lgr5^+^ population, which resides at the apex of its E9.5 hepatoblast hierarchy. Furthermore, we also describe a previously unidentified subpopulation of cholangiocyte-committed cells that do not express Lgr5. To our knowledge, this is the first report that recognizes the functional heterogeneity of the E9.5 hepatoblast pool and the first demonstration that *Lgr5* is a bona fide marker of early bipotent hepatoblasts in the developing liver. Our studies raise further questions about the nature of Lgr5 in liver development and liver morphogenesis. Wnt signalling has been implicated in liver growth (McLin et al., 2007; Micsenyi et al., 2004; Tan et al., 2008); however, its role in determining the potency of hepatoblasts is unknown. Elucidating the functional role, if any, for Lgr5 in liver development could help to clarify the part played by Wnt signalling. However, knockdown experiments may not be sufficient to address the role of Lgr5 due to the presence of other homologues, such as Lgr4, which is expressed during liver development (Camp et al., 2017), and could compensate for the loss of function of Lgr5, as is reported for the adult intestine (de Lau et al., 2011). Therefore, it remains unclear whether Lgr5 per se has a functional role in liver development. Similarly, cell ablation studies would be required to address whether the Lgr5^+^ hepatoblast, rather than *Lgr5* per se, is indeed required during development. Owing to the widespread expression of Lgr5^+^ stem cells in the adult and in other embryonic tissues, assessing the functionality of Lgr5^+^ cells in a specific tissue is not straightforward. We have shown that isolated Lgr5^+^ hepatoblasts can be cultured *in vitro*, and so this may provide a reductionist system in which we can test the requirement for Lgr5 in establishing or maintaining bipotency in hepatoblasts without the confounding effects of signalling from other tissues. Finally, it would be of interest to investigate whether the adult ductal-regenerative response, in which Lgr5 is upregulated in regenerative liver cells, recapitulates the same programmes as embryonic development. Future studies would aim to address these issues.

## MATERIALS AND METHODS

### Mouse strains and animal work

Lgr5-IRES-CreERT2 (Huch et al., 2013), Lgr5-EGFP-IRES-creERT2 (Barker et al., 2007), R26R-TdTomato (Madisen et al., 2010), R26R-Confetti (Snippert et al., 2010), R26R-CreERT2 (Ventura et al., 2007) and R26R-Rainbow1.0 (Livet et al., 2007) mice have been described previously. All mouse experiments were regulated under the Animals (Scientific Procedures) Act 1986 Amendment Regulations 2012 following ethical review by the University of Cambridge Animal Welfare and Ethical Review Body (AWERB) and were performed in accordance with the Home Office license awarded to M.H.

### Tamoxifen induction

Lineage tracing was performed using the R26R-TdTomato, R26R-Confetti or R26R-Rainbow1.0 reporter in combination with a temporally inducible Cre, either Lgr5-IRES-CreERT2 or R26R-CreERT2. To induce Cre activity, tamoxifen (Sigma-Aldrich, T5648) was administered by intraperitoneal injection of the pregnant female at the specified embryonic day. Tamoxifen doses were dependent on the reporter line and Cre line used. For details refer to Table S1, parts 1 and 2. Embryos and pups (male and females) were then collected at specified time-points according to the experiment.

### Tissue preparation and immunostaining

Embryonic and postnatal livers were dissected and fixed for 2 h or 24 h, respectively, in 10% neutral-buffered formalin (Sigma-Aldrich) at 4°C. Postnatal livers were embedded in 4% low melting point agarose (Bio-Rad Laboratories) and sectioned at 100 μm using a Leica VT1000S microtome. E10.5 and E11.5 samples were equilibrated with 30% sucrose, embedded in OCT compound (VWR, 361603E) and frozen on dry ice in preparation for sectioning with a Leica CM-3050S cryostat at 50 µm. To reduce nonspecific staining and permeabilize the sample, samples were incubated with a 2% donkey serum, 1% Triton and 5% DMSO in PBS solution overnight at 4°C. Primary antibodies were then applied at appropriate dilutions for 48 h at 4°C; for details refer to Table S3, part 1. Samples were washed and secondary antibodies applied at a dilution of 1:250 for 48 h at 4°C. Nuclei were counterstained with Hoechst 33342 (1:1000, Invitrogen) for 30 min at room temperature. Details of secondary antibodies are provided in Table S3, part 1.

### Confocal imaging

Samples were imaged on an SP8 White Light inverted confocal microscope (Leica Microsystems) through a 10× or 20× objective using Leica application suite X Software. Optical sections were acquired at 2 µm intervals. Images were processed using Fiji.

### Frequency of merger events

To calculate the frequency of mergers of the same colour we followed the calculations published by Aragona et al. (2017). Briefly, the probability of unicolour mergers is proportional to the frequency of bicolour mergers involving a given colour taking into account different induction frequencies between colours. This analysis was conducted using the livers in which bipotent clones were quantified.

### Automated cell counting of homeostatic proportions

Automated cell counting was conducted on immunofluorescent images (stained for osteopontin and Hoechst) of P0, P14 and P30 liver sections to determine the homeostatic proportions of hepatocytes and cholangiocytes in the postnatal liver. First, images were segmented using ilastik-1.2.2 software. In this way, immunofluorescent images consisting of a Hoechst channel and an osteopontin channel were used to train the machine learning software to segment cholangiocytes (primarily based on co-expression of osteopontin), hepatocytes (primarily based on their characteristic large round nuclei), other cells (primarily based on a lack of osteopontin co-staining and smaller, brighter nuclei) or background (based on a lack of signal). The segmented images were then imported into Fiji and, using the Threshold plug-in, the specific hepatocyte or cholangiocyte segments were selected. A selection was created around the hepatocyte or cholangiocyte segments and overlaid on the Hoechst channel, which clearly shows individual cells. Within the cholangiocyte or hepatocyte selections, the number of cells were counted using the find maxima function on the Hoechst channel.

### AFP and albumin secretion assays

To assess AFP and albumin secretion, the Mouse alpha Fetoprotein ELISA Kit (Abcam) and the Mouse Albumin ELISA kit (AssayPro), respectively, were used according to the manufacturers' instructions (Table S2, part 2). Supernatants were collected 24 h following the most recent medium change.

### Isolation of cells for single cell RNA sequencing and *in vitro* culture

Lgr5GFP^het^ and Lgr5GFP^−/−^ embryos were collected at the specified time-points and screened for GFP signal with an epi-florescence microscope. Once classified according to phenotype, livers were collected and minced before enzymatic digestion. Enzymatic digestion was performed at 37°C with Wash medium [constituting DMEM+ GlutaMAX (Invitrogen) supplemented with 1% FBS and 1×penicillin/streptomycin] containing 0.125 mg/ml collagenase type I (Sigma-Aldrich) and dispase II (Gibco), and 0.1 mg/ml DNase (Sigma-Aldrich). The incubation time for enzymatic digestion was ∼40 min for E10.5 livers and 2 h for E13.5 livers. Once the digestion to single cells was confirmed by visual inspection, samples were filtered through a 40 μm pore nylon cell strainer (Falcon) and centrifuged at 400 ***g*** for 5 min. The pellet was resuspended in blocking solution [Wash medium with 2% FBS, Rho kinase inhibitor Y27632 (Sigma-Aldrich) and 0.1 mg/ml DNase] for 20 min. Cells were then centrifuged at 400 ***g*** for 5 min and incubated with primary antibody against Liv2 (1:100, MBL) in wash medium supplemented with rock inhibitor and DNase for 40 min on ice. Cells were then pelleted at 400 ***g*** for 5 min and washed. Cells were incubated with APC anti-rat (Biolegend) for anti-Liv2, CD31-PE/Cy7 (Abcam) and CD45-PE/Cy7 (Bioscience) diluted in wash medium supplemented with rock inhibitor and DNase for 40 min on ice. The sorting strategy consisted of a population of single cells that were sequentially gated based on cell size (forward scatter, FSC, versus side scatter, SSC), singlets (pulse width versus FSC) and Liv2-APC positivity. Finally, CD45-PE/Cy7 (BD Biosciences) and CD31-PE/Cy7 (Abcam) antibodies were used in order to exclude blood cells and endothelium. Liv2^+^/CD31^−^/CD45^−^ (bulk hepatoblast pool) or Liv2^+^/CD31^−^/CD45^−^/GFP^+^ cells (named Lgr5^+^ cells) were used for further analysis.

For single cell RNA-sequencing experiments, cells were sorted on an influx Cell Sorter (BD Biosciences). Single cells were collected in non-skirted PCR plates containing lysis buffer [0.2% triton (Sigma-Aldrich Triton X-100 solution) in 1U per µl RNase inhibitor (Thermo Fisher Scientific) in DEPC-treated water (Ambion)]. Plates were then vortexed and centrifuged at 370 ***g*** for 2 min and kept at −80°C. For 3D *in vitro* culture, cells were sorted on a MoFlo into Sort medium [Advanced DMEM/F12 (GIBCO) supplemented with 1% penicillin/streptomycin, 1% glutamax, 10 mM HEPES, 1×B27 supplement (without vitamin A), 1.25 mM N-acetyl-l-cysteine, 10% (vol/vol) Rspo-1 conditioned medium, 10 mM nicotinamide, 10 nM recombinant human (Leu15) gastrin I, 50 ng/ml recombinant mouse EGF, 100 ng/ml recombinant human FGF10, 25 ng/ml recombinant human HGF, 1 nM A8301 and 10 µM Y27632].

### Single cell RNA-sequencing

scRNA-seq sample preparation was performed using an adapted version of Smartseq2 (Picelli et al., 2014). cDNA was reverse transcribed using 50 U reaction SmartScribe Reverse Transcriptase (Takara ClonTech) without betaine and MgCl_2_, and amplified using KAPA HiFi Hotstart polymerase (Roche). Illumina Nextera XT DNA preparation kit was used to prepare libraries, and pooled libraries were sequenced using the Illumina HiSeq 4000 system (single-end 50 bp reads). The quality of the reads was examined with FastQC (www.bioinformatics.babraham.ac.uk/projects/fastqc/). The reads were aligned to genome version GRCm38, with the 92 Spike-in transcript sequences added, using STAR (v2.6.0c) and Ensemble gene annotation version 93 (Kersey et al., 2018). Subread (v1.6.2) was used to count uniquely aligned reads using the same Ensemble annotation and to create the count matrix. Further analysis was performed using scanpy (v1.3.3) (Wolf et al., 2018b). For quality control of cells, the following quality metrics were calculated for each cell: (1) the percentage mitochondrial transcript reads, (2) the percentage of spike-in reads, (3) the total number of reads, and (4) the log10 transformed number of genes with at least one read. Only cells with (1) less than 20% of mitochondrial reads, (2) less than 25% spike-in reads and (3) more than 100,000 reads were considered for downstream analysis. As the log10 transformed number of genes with at least one read (4) showed clear batch effects, the four different thresholds 3.6, 3.5, 3.7 and 3.5 were applied to the four different sorts and only cells exceeding these thresholds passed quality control. In total, 653 (69%) of 943 cells were considered for downstream analysis. Because an initial principal component analysis revealed batch effects between the biological replicates from experiments 1, 2 and 4 (group 1) on the one hand and experiment 3 (group 2) on the other hand, batch correction between those two groups was performed: for each group, only genes expressed in at least 3 cells were considered. The counts in each group were normalised using size factors computed with the scran (v1.8.4) function computeSumFactors [parameters: min_mean=1.0, size=seq(20, 100, 5)] (Lun et al., 2016). For each group, highly variable genes were detected using the scanpy function filter_genes_dispersion (parameter: max_mean=8) and the intersection of both gene sets, which contained 1766 genes, was used for further analysis. Batch effects between the two datasets were corrected by matching mutual nearest neighbours in the implementation of mnnpy (v0.1.9.3) (parameters: svd_mode=‘irlb’) (Lun et al., 2016). On the resulting count matrix, a principal component analysis was performed. t-SNE dimensionality reduction was performed on the first 20 principal components using the MulticoreTSNE implementation (parameters: perplexity=80, early_exageration=12) (Amir et al., 2013). To perform Louvain clustering, the 15 nearest-neighbours graph was computed on the first 20 principal components. Using Louvain clustering with the resolution parameter set to 0.05, three clusters were obtained (Levine et al., 2015; Šubelj and Bajec, 2011). Differentially expressed genes were detected by performing a Wilcoxon rank-sum test on the raw counts comparing each cluster against the union of the other two clusters as implemented in scanpy's rank_genes_groups function. To define marker genes for the clusters at specific embryonic stages, the cells were grouped according to cluster and stage, and a Wilcoxon rank-sum test was performed as described above. For the sub-clustering of the cholangiocyte-like cluster, a principal component analysis was performed on those cells and then clustering was performed as above with the resolution parameter set to 0.5. Differentially expressed genes between the two resulting sub-clusters were detected as described above. The diffusion maps were calculated using the scanpy function diffmap with the width of the Gaussian connectivity kernel being implicitly determined by the distance to the 100 nearest neighbours in the space of the 20 first principal components (Coifman et al., 2005; Haghverdi et al., 2015). Diffusion pseudotime was calculated using scanpy's dpt function using the cell with minimal diffusion component 1 as root cell (Haghverdi et al., 2016; Wolf et al., 2018a). Cell cycle phases were assigned using cyclone and the pre-trained mouse cycle markers contained in the scran package (Scialdone et al., 2015). Cells were classified as Lgr5 positive at the transcript level if they had more than 10 reads of Lgr5. The code is available on GitHub at github.com/fabianrost84/prior_et_al_2019.

### 3D culture of embryonic liver cells

Following isolation, as described in the isolation section above, the cells were pelleted at 400 ***g***, seeded in Matrigel (BD Biosciences) and cultured either with the hepatocyte-like protocol or cholangiocyte-like protocol. The hepatocyte-like method involves culturing for the first 3 days in Advanced DMEM F12 supplemented with 1% penicillin/streptomycin, 1% glutamax and 10 mM HEPES (Gibco), 1×B27 (Gibco), 1.25 mM n-acetylcysteine (Sigma-Aldrich), 10 mM nicotinamide (Sigma-Aldrich), 100 ng/ml FGF10 (Peprotech), 100 ng/ml FGF7 (Peprotech), 50 ng/ml HGF (Peprotech), 10 nM gastrin (Sigma-Aldrich), 50 ng/ml EGF (Peprotech), 1 nM A83-01 (Tocris Bioscience), 3 µM CHIR 99021 (Tocris Bioscience), 15% (vol/vol) Rspo-1 conditioned medium (in house) and 10 µM Rock inhibitor Y-27632 (Sigma-Aldrich). From day 3 onwards, the culture medium was modified by the exclusion of FGF7. The cholangiocyte-like method consists of culturing for the first 3 days in Advanced DMEM F12 supplemented with 1% penicillin/streptomycin, 1% glutamax and 10 mM HEPES 1×B27, 1.25 mM n-acetylcysteine, 10 mM nicotinamide, 100 ng/ml FGF10, 50 ng/ml HGF, 10 nM gastrin, 25 ng/ml noggin (Peprotech), 50 ng/ml EGF, 1 nM A83-01, 10 µM forskolin (Tocris Bioscience), 10% (vol/vol) Rspo-1 conditioned medium, 30% (vol/vol) Wnt conditioned medium (in-house) and 10 µM Rock inhibitor Y-27632 (Sigma-Aldrich). From day 3 onwards, the culture medium was modified by the exclusion of the Wnt conditioned medium and removal of noggin. After several days in culture, organoid structures with either a cystic (cholangiocyte-like medium) or solid (hepatocyte-like medium) form arose. Cultures were split in a 1:2 ratio after 14-20 days.

## Supplementary Material

Supplementary information
