# Supplementary material for: Lgr5+ stem and progenitor cells reside at the apex of a heterogeneous embryonic hepatoblast pool
Source: Development. 2019 Jun 12;146(12):dev174557. doi: 10.1242/dev.174557 (PMC6602348; doi:10.1242/dev.174557)
Supplement: Supplementary information [file develop-146-174557-s1.pdf]

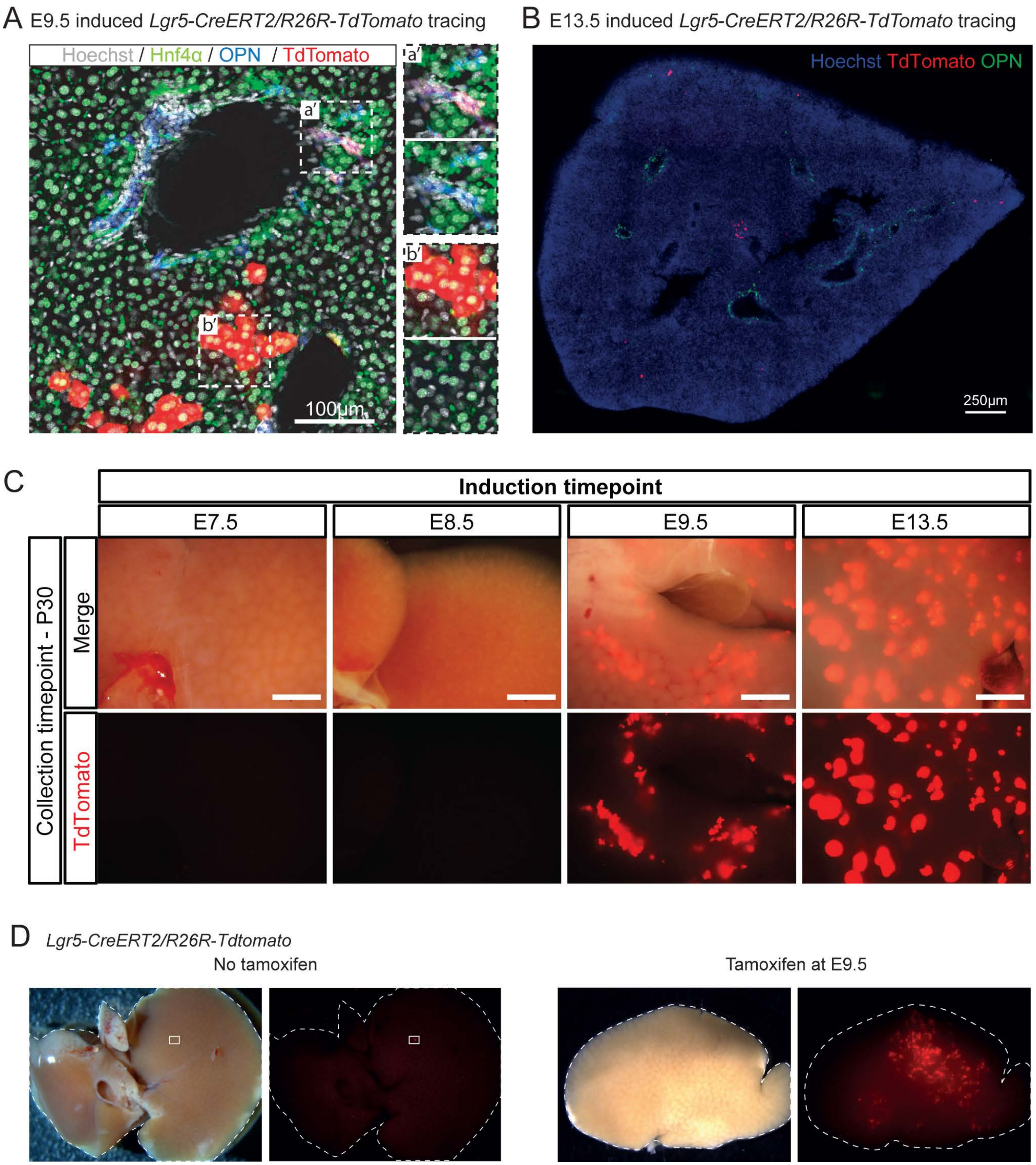


**Fig. S1: Lgr5 labels hepatoblasts from E9.5 to at least E13.5 of liver development.** (A-D) *Lgr5-ires-CreERT2$^{hom}$; R26R-TdTomato$^{hom}$* males were mated with MF1-WT females in order to generate the compound mice *Lgr5-IRES-CreERT2$^{het}$;R26R-*

Development • Supplementary information

*TdTomato*$^{het}$. (A) Induction of lineage tracing at E9.5 leads to labelled cholangiocytes (co-stained with OPN) and hepatocytes (co-stained with Hnf4α), liver collected at P30. Magnifications of labelled cholangiocytes and hepatocytes are shown in a') and b'), respectively, upper panels include TdTomato signal, lower panels exclude TdTomato signal to clearly demonstrate the type of cell labelled. (B) Induction of lineage tracing at E13.5 resulted in hepatocyte progeny only. (C) Cre activity was induced at the indicated timepoints and livers collected at P30. Expression of TdTomato was detected in postnatal livers only if induction was at E9.5 or later, suggesting that Lgr5 is expressed in the developing liver from E9.5 onwards. Scale bar = 2mm. (D) TdTomato tracing was almost never detected in non-tamoxifen induced controls. Only in one mouse we detected <100 cells labelled in the liver. In contrast, the tracing events in the livers of the tamoxifen induced embryos was clear with several thousands of cells labelled. Both the non-induced and induced livers were collected at P14. This minute number of labelled cells in the non-induced livers does not affect our interpretations in the lineage tracing experiments.

Development • Supplementary information

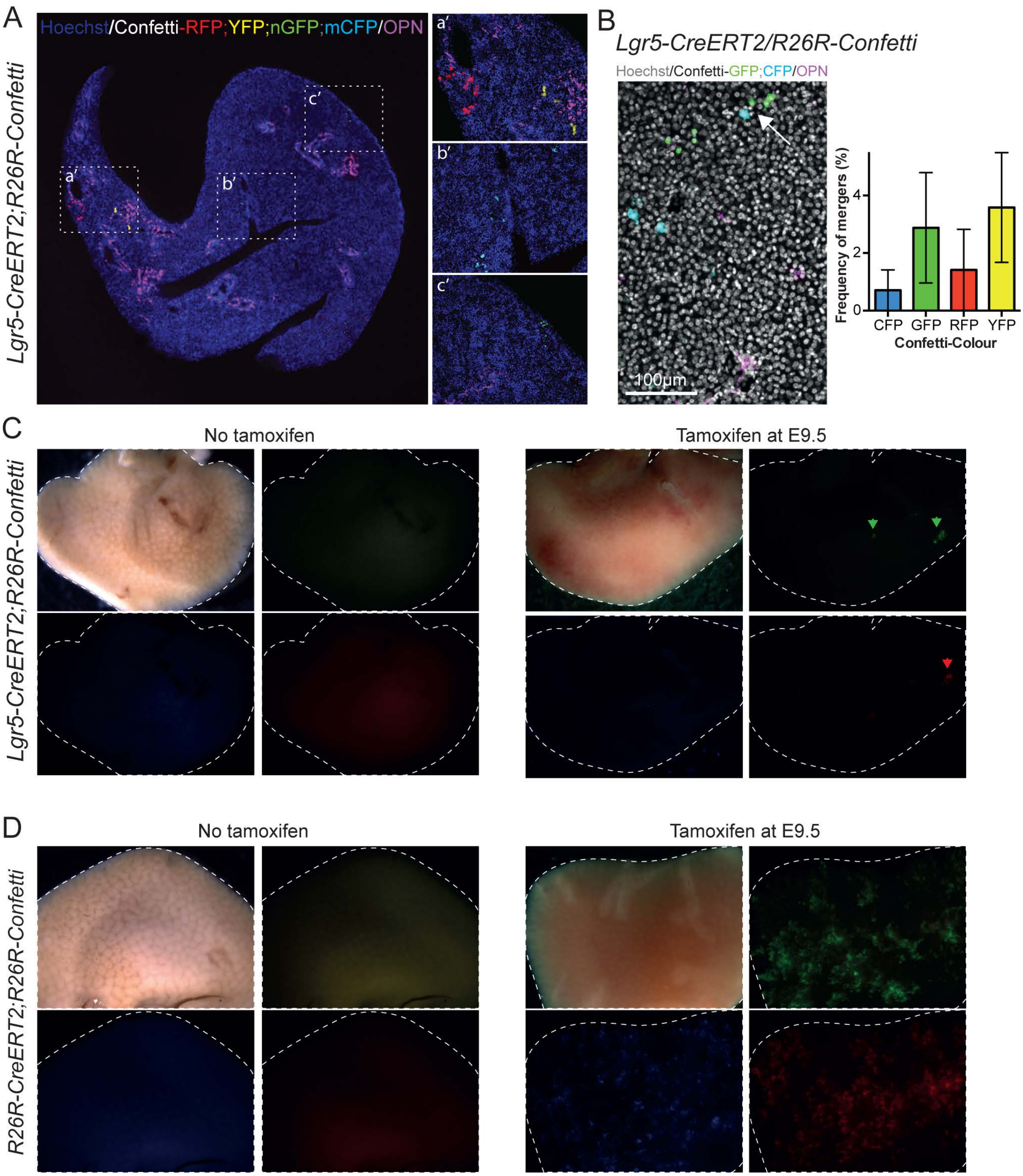


**Fig. S2: Lineage tracing from Lgr5-IRES-CreERT2;R26R-Confetti and R26R-Cre;R26R-Confetti mice induced at E9.5.** (A-C) *Lgr5-IRES-CreERT2$^{het}$;R26R-Confetti$^{het}$* mice were generated by breeding *Lgr5-IRES-CreERT2$^{hom}$* with the multicolour Confetti reporter *R26R-Confetti$^{hom}$* and liver tissues were collected postnatally. (A) Labelling

Development • Supplementary information

of the E9.5 embryonic livers of *Lgr5-IRES-CreERT2$^{het}$;R26R-Confetti$^{het}$* mice results in cells labelled in one of four colours (RFP, YFP, mCFP, nGFP). A representative image of a liver section presenting clones in each of the 4 colours is shown. Tissue was co-stained with Osteopontin to visualize the ductal cells (OPN, magenta). Nuclei were counterstained with Hoechst. a') magnified area showing a red and a yellow clone. b') magnified area showing a mCFP clone. c') magnified area showing a nGFP clone. Note that no merging of clones is observed. (B) Bicolour-merger events, *i.e.* clones of different colours merging, were rarely detected. Representative image of one of the 5 merging events observed in the n=3 livers analysed for bipotent clones (n=2, liver_1; n=3, liver_2, n=0, liver_3). Example of a merging event between a CFP and GFP clone following induction at E9.5 in Lgr5-IRES-CreERT2$^{het}$;R26R-Confetti$^{het}$ embryos. Graph represents the mean ± SEM of the frequency of mergers within the same colour. (C-D) Tracing events from the R26R-Confetti reporter in combination with the *Lgr5-IRES-CreERT2* (C) or *R26R-CreERT2* (D) driver only occur upon tamoxifen administration and are never found in non-induced mice.

Development • Supplementary information

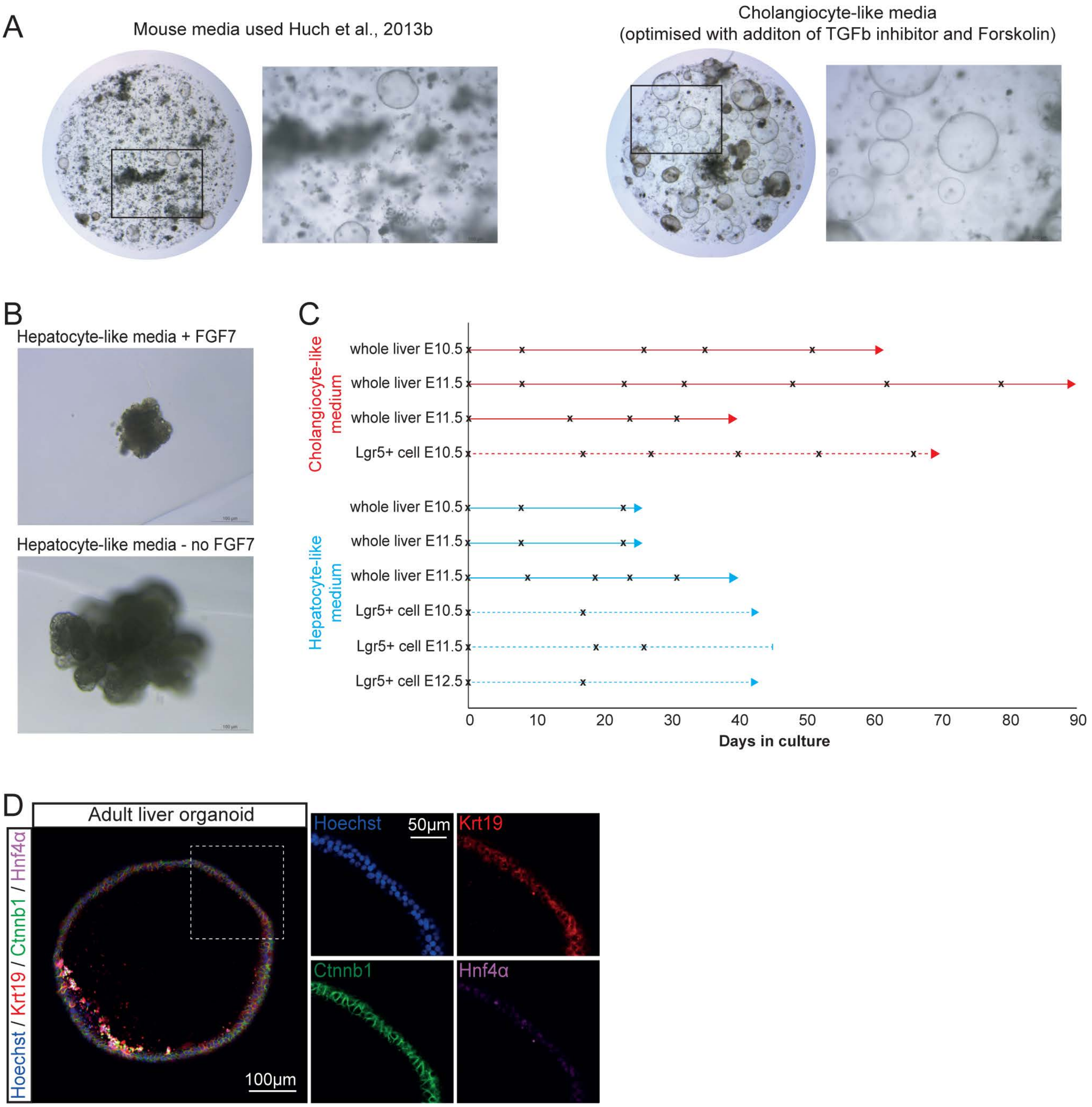


**Figure S3: Generation of mouse embryonic liver organoids.** (A-B) Embryonic liver organoids were generated from whole liver tissue obtained from E11.5 *WT* embryos dissociated as described in methods. (A) Isolated cells were embedded in Matrigel and cultured in our mouse adult ductal liver organoid medium (Huch et al., 2013) containing EGF, Noggin, Rspondin1, FGF10, HGF, Nicotinamide (left panel) or in the same medium supplemented with 1 nM A8301 and 10 μM Forskolin (optimized Cholangiocyte-like medium,

Development • Supplementary information

right panel) as described in methods. This resulted in an evident increase in the number and size of organoids formed. (B) Isolated cells embedded in Matrigel were also cultured in the recently published hepatocyte medium that sustains human embryonic liver growth in vitro (Hu et al., 2018). Removal of FGF7 resulted in a significant improvement on the expansion of the organoids. For details refer to methods. (C) Comparison of renewal potential between embryonic organoids derived from 'whole liver tissue' or 'sorted Lgr5+'cells. We find comparable renewal potentials between 'sorted Lgr5+' and 'whole liver tissue' cholangiocyte-like organoids. In the case of the hepatocyte-like organoids the 'whole liver tissue' cells form structures which can be easily passaged (up to P4 to date). Although, the 'sorted Lgr5+' cells readily form hepatocyte-like organoids and can withstand initial passaging, their viability then decreases. (D) Immunofluorescence staining for Ctnnb1 with the ductal marker Krt19 and the hepatocyte marker HNF4α in adult ductal liver organoids cultured in our standard organoid medium as described in (Huch et al., 2013). These served as positive control for the stainings shown in Figure 4.

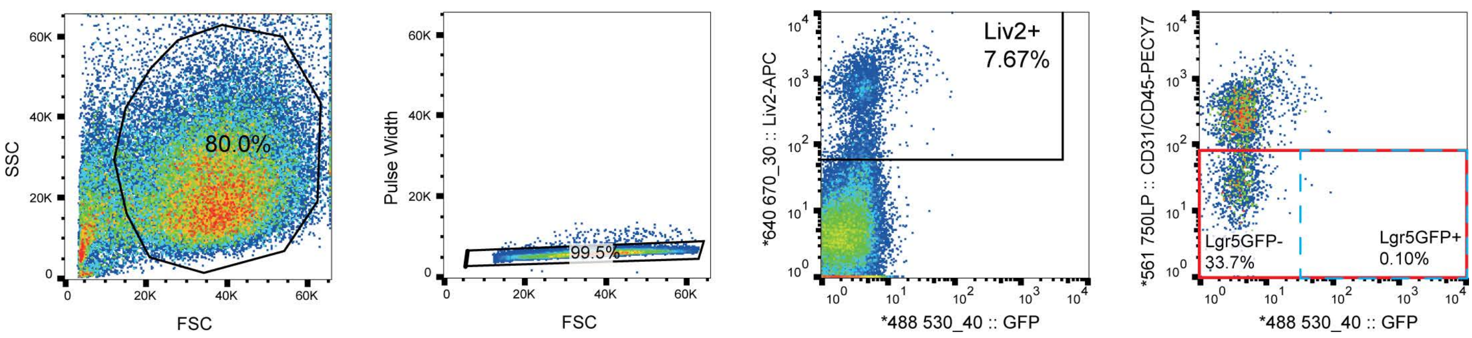


**Figure S4: Lgr5+ hepatoblast sorting strategy.** *Lgr5-EGFP-IRES-CreERT2$^{het}$* mice were mated with MF1-WT females and embryos collected at E10.5 of gestation. WT and *Lgr5-EGFP-IRES-CreERT2$^{het}$* littermate embryos were scored for the presence of eGFP in the cranial area. Then, embryos were split by genotype and liver tissues collected and processed for cell isolation and single cell dissociation. Cells were stained with the hepatoblast marker Liv2, the endothelial maker CD31 and pan-haemopoietic marker CD45 as described in methods. Sorted cells were obtained following a sequential gating strategy where cells were first gated by FSC vs SSC, then FSC vs Pulse width to identify singlets and then gated for Liv2+ (bulk hepatoblasts, Liv2+CD31-CD45- (red box)) or Liv2+GFP+ (Lgr5+ hepatoblasts, (Liv2+CD31-CD45-GFP+ (blue dashed box))).

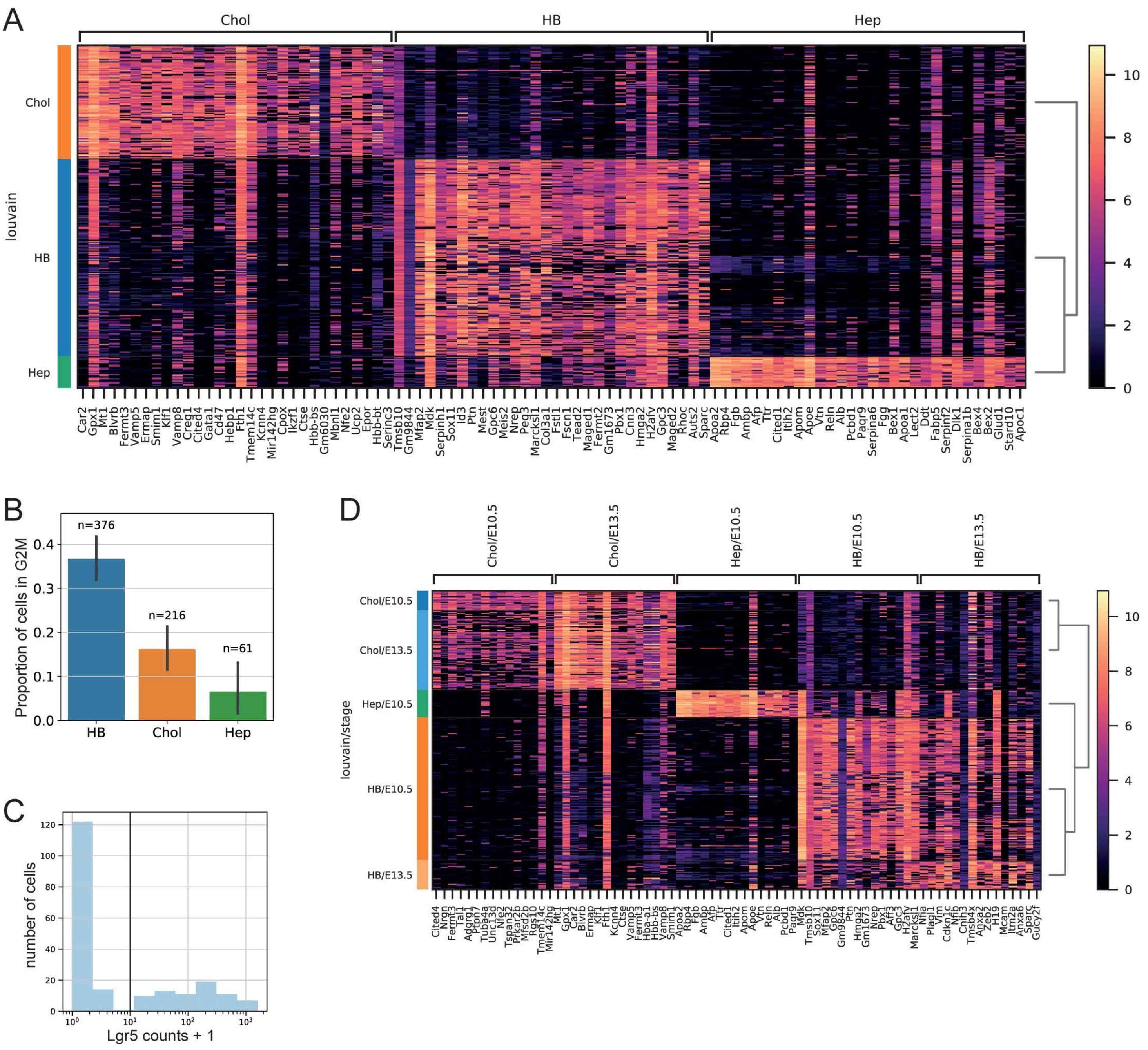


**Figure S5: scRNA-seq of hepatoblasts reveals heterogeneity in the hepatoblast population.** (A) Heatmap displaying the 30 most differentially expressed genes in each cluster as detailed in Supplementary Table 2, (colour represents the log-transformed number of reads (with an offset of 1), dendograms represent hierarchical clustering of cells). (B) Proportion of cells in G2M phase of the cell cycle in the hepatoblast cluster (HB), cholangiocyte-like cluster (Chol) and hepatocyte-like cluster (Hep) (mean±95% confidence intervals). (C) Histogram of the distribution of Lgr5 counts shows a bimodal distribution. A threshold of 10 counts is used to define a cell as Lgr5+ on the transcript level. Using this threshold, we find that 2% of the bulk cells at E10.5 are Lgr5+ at the transcript level.(D) Heatmap displaying the differentially expressed genes by time point, (colour represents the log-transformed number of reads (with an offset of 1), dendograms represent hierarchical clustering of cells). For extended list see Supplementary Table 2_part 2-part 6.

Development • Supplementary information

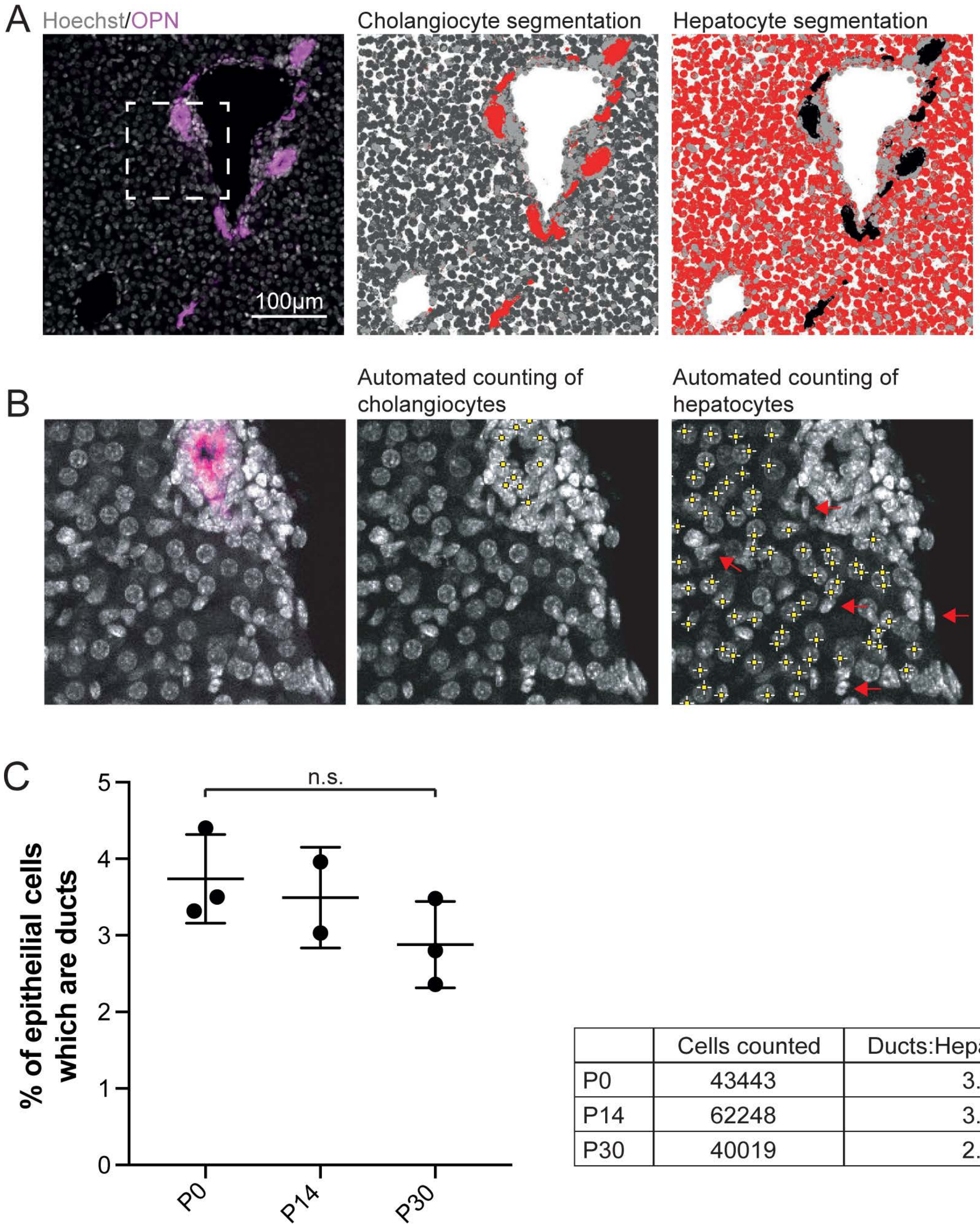


| | Cells counted | Ducts:Hepatocytes |
|---|---|---|
| P0 | 43443 | 3.72% |
| P14 | 62248 | 3.55% |
| P30 | 40019 | 2.91% |

**Figure S6: Counting of hepatocyte and cholangiocyte cell proportions in the homeostatic postnatal liver.** (A-C) To determine the homeostatic proportions of hepatocytes and cholangiocytes at the different postnatal time points of interest osteopontin (OPN) staining was performed to mark the ductal cells and then the proportion of hepatocytes vs cholangiocytes was counted using an automated system. (A) Osteopontin (OPN, purple) marks ductal cells in the P14 liver (nuclei are counter stained with Hoechst 33342). Immunofluorescent images were segmented using ilastik-1.2.2 software, machine learning was used to train the software to segment cholangiocytes (middle panel), hepatocytes (right panel), other cells or background, segmented cells are marked in red). The segmented images were then imported into Fiji, a selection was created around the segmented images and overlaid on the Hoechst channel. Within the cholangiocyte or hepatocyte selections the number of cells were counted using the find maxima function on the Hoechst channel. (B) Magnification of A) to show the cells counted (yellow cross-hairs) following segmentation of cholangiocyte and hepatocyte cells. Note only epithelial cells are counted, other cells including mesenchymal and endothelial cells (a selection are highlighted with red arrows) are not counted. (C) Automated counting reveals the homeostatic number of cholangiocytes as a percentage of epithelial cells is ~3% at all 3 time points (P0, P14 and P30) analysed. Graph represents the percentage of cholangiocytes within the total epithelial cells counted at the 3 time points analysed (mean ± STDEV). On average a 3.4% ± 0.6% of epithelial cells of the mouse liver are cholangiocytes, this proportion does not change over postnatal days P0 - P30 (mean ± STDEV). Table indicates the total number of cells counted and the % of cholangiocytes within these at each of the 3 time points analysed.

Development • Supplementary information

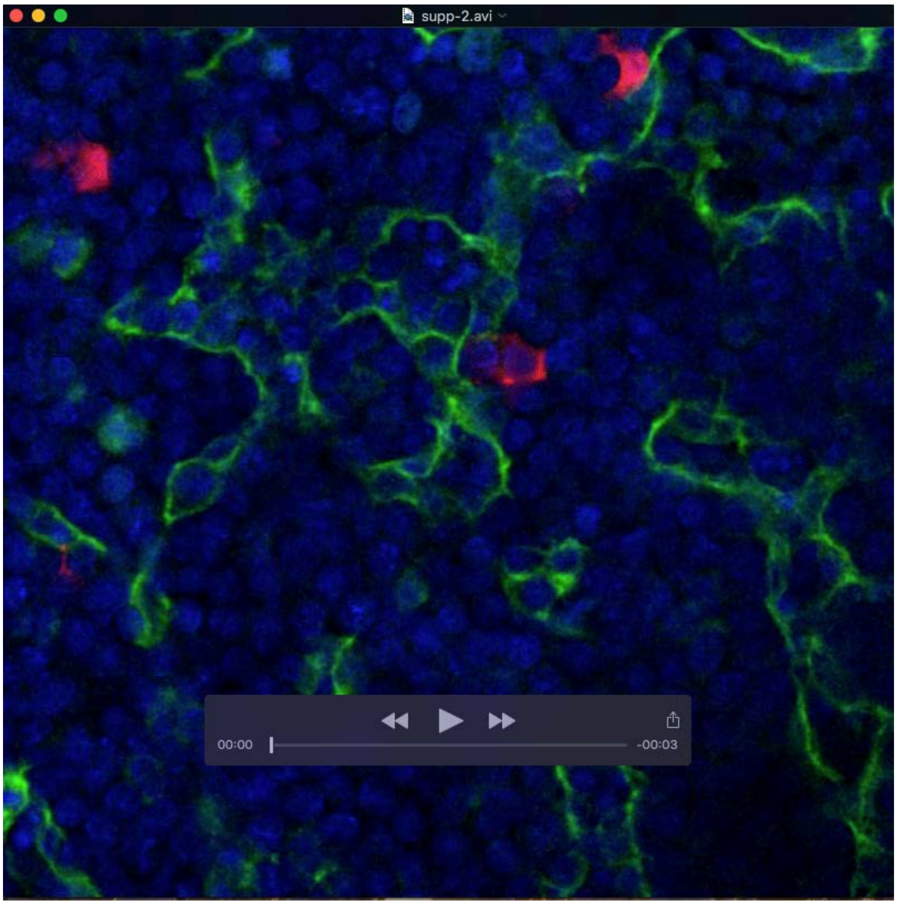


**Movie 1: Confocal Z-stack video showing co-staining of TdTomato+ Lgr5+ descendants with the endothelial marker VEGFR3 at E11.5 following induction at E9.5**

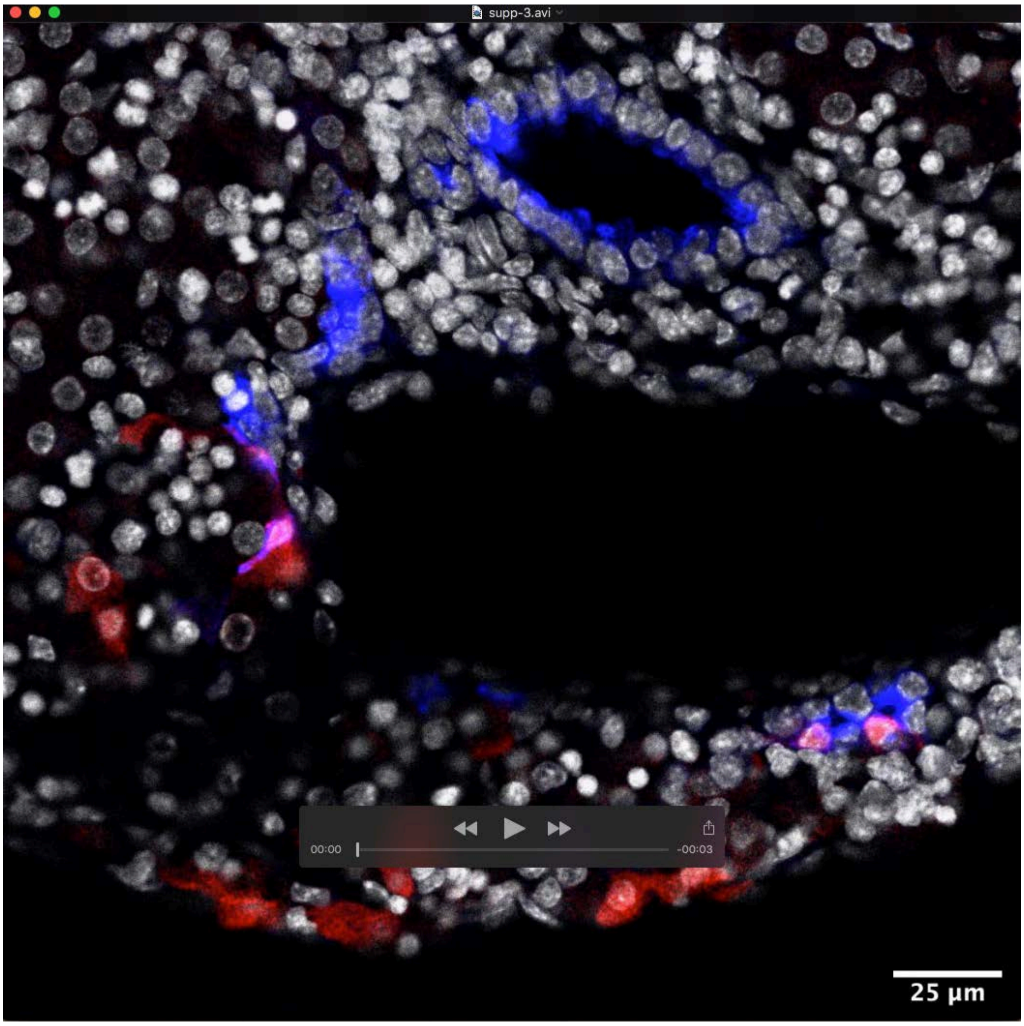


**Movie 2: Confocal Z-stack video of a bipotent Lgr5 clone derived from an *Lgr5-IRES-creERT2;R26R-Confetti* embryo injected at E9.5**

**Table S1. Tracing counts used for the analysis**

Click here to Download Table S1

**Table S2. scRNA-seq gene lists**

Click here to Download Table S2

**Table S3. List of materials and reagents used in the manuscript**

Click here to Download Table S3
